# Attitudes of Longitudinal Study Participants and Members of the Public Towards Data Sharing for Secondary Research: A Survey Based Approach

**DOI:** 10.1177/15562646261452724

**Published:** 2026-06-03

**Authors:** Nicola Howe, Thomas Chadwick, Dorothy Newbury-Birch, Elaine McColl

**Affiliations:** 1 NIHR HealthTech Research Centre in Diagnostic and Technology Evaluation, 5994 Newcastle University, Newcastle upon Tyne, NE2 4HH, UK; 2 Population Health Sciences, Faculty of Medical Sciences, 5994 Newcastle University, Newcastle upon Tyne, NE2 4AX, UK; 3 SSSHL Department of Law, Policing and Investigation, 5462 Teesside University, Middlesbrough, TS1 3BX, UK

**Keywords:** Data sharing, participants, longitudinal study, informed consent, secondary use, patient perspective, ALSPAC

## Abstract

Most research funders and journals now require researchers to make their data available for sharing. There is a growing body of literature on research participants’ attitudes towards health data sharing, but less evidence regarding views of participants taking part in longitudinal studies, clinical trials or public health research.

1,664 respondents from the UK (participants in longitudinal studies ALSPAC and ACONF and members of Patient and Public Involvement (PPI) groups completed a questionnaire survey exploring attitudes towards data sharing, including consent and data storage. Respondents were most concerned about privacy and data security and highlighted concerns about open access and sharing with commercial organisations.

## Introduction

The benefits of ‘Data Sharing’, the process by which anonymised (identifying or personal information removed) research data are made available for utilisation by other researchers, are well extolled by funders, journals and research-supporting organisations (Chawinga & Zinn, 2019; Editorial, 2018; Godlee & Groves, 2012; Loder, 2013; Loder & Groves, 2015; OECD, 2007; PLOS, 2014; Ross & Krumholz, 2013; Taichman et al., 2016; Walport & Brest, 2011). One of the most oft-cited benefits is the chance for researchers to make use of data already collected to conduct their own ‘secondary research’ unrelated to the primary study (Kelly et al., 2024), thereby increasing research efficiency, providing greater benefits to science and ultimately leading to improved or more rapidly discovered treatments for patients (Mello et al., 2013). Data sharing is also said to increase trust in research, through transparency both in the research process, the results and in use of data (both how and why) (Aitken et al., 2016b; Loder, 2013; Waind, 2020). Trust is ever more important in a changing research landscape in which the introduction of the General Data Protection Regulation (GDPR) (Information Commissioners Office, 2019; Vlahou et al., 2021) brought to light that participants have an increasing awareness of their rights to control use of their personal data (Hirst et al., 2023; Shah et al., 2018). Getting the most out of participants’ data in theory means that individuals will need to take part in fewer original studies, reducing the level of risk to which they are exposed (Mello et al., 2013; Shabani & Obasa, 2019; Vallance et al., 2016). In the spirit of transparency, datasets collected using public and charitable funds in the public interest should also release their data for public scrutiny (Attwood & Munafò, 2016; Borgman, 2012; Carr & Littler, 2015; Institute of Medicine (IOM), 2015; Institute of Medicine (US), 2013; Mello et al., 2013; UKRI, 2025).

Researchers have explored the ways in which the research community may be encouraged to share their data (Borgman, 2012; Bouter, 2016; Hajduk et al., 2019; Koers, 2016; Mauthner & Parry, 2013; Ohmann et al., 2017; Prisco et al., 2016; van Panhuis et al., 2014) but historically less attention has been paid to the ways in which participants might be encouraged to *agree* to share their data (i.e., to patient or participant attitudes).

In literature exploring attitudes towards data sharing ‘health’ data may be used to refer to various types of data including routinely collected hospital or GP electronic health record data (diagnoses, treatments and referrals), clinical trial data (clinical history and responses to treatments), biobank data (samples such as blood, or saliva, detailed health and genetic information) or linkage of differing data sets using a common identifier. Many studies of participant attitudes tend to combine these different types of health data (Hill et al., 2013; Hutchings et al., 2020; Kalkman et al., 2022; Sánchez et al., 2023). This may be because there are not enough papers in each distinct area, or because researchers assume that participants’ attitudes will be similar regardless of the setting or type of data shared.

Previous empirical studies exploring attitudes towards sharing of ‘health’ data for secondary research have generally concluded that individuals are willing to share their data for altruistic reasons or to improve healthcare (Courbier et al., 2019; Hutchings et al., 2023; Köngeter et al., 2022; Mazor et al., 2017; Stone, Redsell et al., 2005; Yusuf et al., 2024) but that they have concerns about data security, privacy and the potential for exploitation (Baines et al., 2024; Hutchings et al., 2023; Mazor et al., 2017; Yusuf et al., 2024) leading them to desire a certain amount of control over use of their data (Cervera de la Cruz & Shabani, 2023; Courbier et al., 2019) such as consent (sought in advance) for secondary use, at least as a courtesy (Biasiotto et al., 2023; Nair et al., 2004; Stone et al., 2005). Participants are reportedly more hesitant about sharing with commercial or profit-driven organisations or with organisations that they would not have chosen themselves, than with universities for example (Biasiotto et al., 2023; Courbier et al., 2019; Kim et al., 2015; Tosoni et al., 2022). Similar findings have been observed in studies exploring attitudes to linkage of data sets (Aitken et al., 2016b; Audrey et al., 2016; Clarke et al., 2021; Wild et al., 2023; Xafis, 2015) and sharing of clinical, electronic health records and genomic data (Chan et al., 2012; Garrison et al., 2016; Hill et al., 2013; Kalkman et al., 2019; Lemke et al., 2010; Milne et al., 2021; Shabani et al., 2014; Yusuf et al., 2024).

A key aspect of studies exploring attitudes towards sharing is consent (Audrey et al., 2016; Garrison et al., 2016; Kalkman et al., 2019). Previous research is divided as to whether a single consent for sharing at the outset of a study is sufficient or acceptable (Braun et al., 2014; Clerkin et al., 2013; Prisco et al., 2016) or whether a re-consent is required prior to each instance of sharing, a sort of ‘active consent relationship’ renewed over time (Lemke et al., 2010; Ludman et al., 2010; Wild et al., 2023). Electronic dynamic consent models allow participants to monitor use of their data and update consent preferences throughout their lifetime (Prinsen, 2024). Re-consent might also be conditional, depending on the nature of the organisation with which data are to be shared (Grande et al., 2013; Hill et al., 2013; King et al., 2012; Trinidad et al., 2010).

Less well explored is the concept of storage and access to participant data that may be shared, for example from repositories, and who should have access. Data which are open access and have no restrictions placed on access for secondary use are often contrasted with data to which there is controlled access, i.e., where conditions are in place to control who can access it, and what secondary research can be conducted. Shabani et al. identified that control is important to participants: “*people have a right to control their information. It doesn’t matter whether anything bad would happen”* (Shabani et al., 2014 p.6). Researchers themselves have advocated for controlled access (Sydes et al., 2015; Tucker et al., 2016; Tudur Smith et al., 2015).

Despite a growing international body of research on attitudes towards various types of health data sharing which we have outlined above, there is still (with some exceptions: (Audrey et al., 2016; Hutchings et al., 2020; Hutchings et al., 2023; Mello et al., 2018)) a general lack of research focussing specifically on the views of participants in clinical trials, public health research or longitudinal studies. Therefore, the overall focus of this study was research participants’ attitudes towards sharing of data collected as part of clinical trials and health research studies (longitudinal studies or public health research studies). This health data would be typically (but not exclusively) quantitative, collected via measurements or surveys conducted as part of research studies. We hoped also to increase evidence on attitudes towards sharing processes such as consent and storage.

Three key research questions (RQ) were established: RQ1. What are research participants’ attitudes towards data sharing, RQ2. Does knowing about sharing affect the likelihood of taking part in studies and RQ3. What are preferences for sharing processes or procedures?

## Methods

### Study Design

An online questionnaire survey was distributed to participants between October 2019 and July 2020.

### Inclusion Criteria for Participants

The target population for this survey was (a) individuals who had taken part (or were still taking part) in public health research, clinical trials with a public health benefit, longitudinal studies or health interventions within the United Kingdom and might therefore be expected to have views on research data sharing and (b) interested members of the public (who could potentially take part) in such studies. Participants also needed to: be aged over 18; have capacity to give informed consent to take part; be resident in the UK (or taking part in a study that originated in the UK, if current place of residence unknown). Participation was limited to participants resident in the UK so that results might be more applicable to local health services, data access procedures and legislation.

### Recruitment

Non-probability (or convenience) sampling was used and speculative contact was made with 64 authors or investigators of trials or published studies that fitted the eligibility criteria. Of the investigators who responded (n = 29), the most frequent response was that they did not have explicit consent or ethical approval to re-contact participants (n = 12), had no means of re-contacting participants (n = 7), did not wish to over-burden them with further research (n = 6) or had their own plans for re-contact (n = 4). Some responded with encouragement or offered future collaboration. Five responses progressed as far as formal applications that were then rejected due to participant burden. Some investigators (n = 35) did not respond at all, even if followed up. Given the focus of the research, the first two reasons given were, of themselves, interesting and informative.

Despite the initial difficulty in obtaining permission to contact study participants, two studies agreed that their participants would be suitable for contact. These studies were: The Aberdeen Children of the 1950s longitudinal study (Batty et al., 2004) and the Avon Longitudinal Study of Parents and Children (ALSPAC) (Boyd et al., 2013; Fraser et al., 2013). To recruit interested members of the public, three appeals to health-related Patient and Public Involvement (PPI) groups (PRIME Centre Wales, 2018; SAIL Databank, 2020; VOICE, 2017) were also successful. Further details on the included studies, and PPI groups, is given below.

Avon Longitudinal Study of Parents and Children (ALSPAC) (Boyd et al., 2013; Fraser et al., 2013) is comprised of pregnant women resident in Avon, UK with expected dates of delivery between 1st April 1991 and 31st December 1992 who were invited to take part in the study. The initial number of pregnancies enrolled was 14,541 and 13,988 children who were alive at 1 year of age. When the oldest children were approximately 7 years of age, an attempt was made to bolster the initial sample with eligible cases who had failed to join the study originally. The total sample size for analyses using any data collected after the age of seven is therefore 15,447 pregnancies, resulting in 15,658 foetuses. Of these 14,901 children were alive at 1 year of age. 14,203 unique mothers were initially enrolled in the study. As a result of the additional phases of recruitment, a total of 14,833 unique women (G0 mothers) enrolled in ALSPAC as of September 2021.

12,113 G0 partners have been in contact with the study by providing data and/or formally enrolling when this started in 2010. 3,807 G0 partners are currently enrolled. Please note that the study website contains details of all the data that is available through a fully searchable data dictionary and variable search tool: http://www.bristol.ac.uk/alspac/researchers/our-data/. Aberdeen Children of the 1950s is an ongoing study cohort made up of 12,150 participants (6,276 males, 5,874 females) born in Aberdeen between 1950 and 1956, who originally took part in the Aberdeen Child Development Survey, completed in local primary schools in 1962 (Batty et al., 2004).

VOICE is a patient and public involvement (PPI) group based in the UK National Innovation Centre for Ageing (NICA) and was founded in 2007 (VOICE, 2017). Although VOICE is based in the North East of England, its members are spread geographically throughout the UK and in January 2020 VOICE announced that they had joined with Imperial College London. VOICE members provide ideas and feedback on research activity, but also to businesses, charities and community members (VOICE, 2017).

SUPER (Service Users for Primary and Emergency care Research) group members are from diverse backgrounds within Wales, recruited by PRIME Centre Wales (http://www.primecentre.wales/) to support and give patient and public perspectives on research activity focussed upon primary and emergency care, in particular research development and dissemination (PRIME Centre Wales, 2018).

The SAIL Databank PPI group (SAIL consumer panel) was established in 2011 to provide the Welsh public's perspective on research into data linkage in areas such as safeguarding and ethical approval, and to provide input on projects from bid to approval and dissemination stage (SAIL Databank, 2020).

### Ethics

The study was approved by the Newcastle University Faculty of Medicine Ethics Committee (7719/2018) and separately by the trial steering group for Aberdeen Children of the 1950s. Ethical approval for the study was obtained from the ALSPAC Ethics and Law Committee and the Local Research Ethics Committees. Informed consent for the use of all ALSPAC data collected was obtained from participants following the recommendations of the ALSPAC Ethics and Law Committee at the time. Participants can contact the study team at any time to retrospectively withdraw consent for their data to be used. Study participation is voluntary and during all data collection sweeps, information was provided on the intended use of data. When participants followed the link to the online questionnaire survey, they were presented with a participant information sheet and informed that their consent was assumed upon submission of the questionnaire. No identifying information was collected but participants were asked if they would be willing to provide their postcode, with the option to leave this field blank.

### Distribution

An electronic invitation to take part was used for all included groups and the survey was designed in Qualtrics (www. qualtrics.com) with an ‘anonymous link’ to access the survey. This removed the risk of identification of participants but precluded the use of targeted reminders.

The ACONF study manager distributed the invitation and survey link to 1400 participants who were registered to receive a mass email. ALSPAC agreed to distribute the survey link to original cohort members (G1) of ALSPAC whose years of birth were between 1990–92 (excluding those flagged as deceased, withdrawn, or who declined questionnaires or contact; n = 5,858). As is usual for ALSPAC, study data were collected and managed using REDCap electronic data capture tools hosted at the University of Bristol (Harris et al., 2009). REDCap (Research Electronic Data Capture) is a secure, web-based software platform designed to support data capture for research studies. The authors had no access to the survey responses until data collection was complete.

An advert containing the invitation and anonymous survey link was placed on the VOICE website and distributed to approximately 3000 members (which excluded those choosing not to receive ‘invitation to take part’ mailers or the newsletter). All members (approx. n = 30) of SAIL and SUPER groups were sent by email the invitation and anonymous survey link via a fellow (PPI) member of the UK Clinical Research Collaboration (UKCRC) (https://www.ukcrc.org/) data sharing group of which an author (NH) was a member.

### Measures

A systematic review by the authors (Howe et al., 2018) and subsequent scoping focus group conducted with Voice PPI members identified key themes regarding attitudes to data sharing to be included in the questionnaire. Further PPI work was undertaken in the form of cognitive interviewing as described by Willis (Willis, 2005; Willis & Lessler, 1999) to refine the survey questions. Finally, the questionnaires were readability tested by the lead author (NH) (National Learning and Work Institute (England and Wales), 2019; Scott, 2017). Each section of the questionnaire was rated separately with an average overall SMOG score of 15.9, the equivalent of Adult Literacy Standard Level 2 (equivalent to GCSE grades A*-C) (National Learning and Work Institute (England and Wales), 2019) and average Scott readability consensus of reader age: 13–15 years old (Scott, 2017). Closed questions, with multiple-choice response formats, were chosen to minimise respondent burden. As with previous questionnaires exploring attitudes to data sharing (e.g.,: (Mello et al., 2018)), five-point Likert-type scales were chosen for attitude questions, while categories for demographic questions were based upon those used by the Office for National Statistics (Nomis official labour market statistics, 2014; Office of National Statistics, 2016). A final free text question asked respondents if they had any additional comments about data sharing or the survey. The survey had a total of 30 questions split into four sections: taking part in research, attitudes towards sharing, preferences for sharing procedures and participant characteristics. Explanatory text was provided giving definitions of data sharing, consent and storage. Respondents were reminded that data would be anonymised prior to sharing. We also defined what we meant by clinical trials and health research studies and the type of data that might be collected. We deliberately avoided references to data linkage, routinely collected data or genomic data as these were outside of the scope of this survey. The questionnaire is provided in Supplementary materials.

### Analyses

Analyses were conducted in Stata version 15 (https://www.stata.com/stata15/). Due to the anonymous survey link, missing or contradictory answers could not be checked and corrected. Instead, the questionnaires in both Qualtrics and in REDCap (for the ALSPAC collection) were constructed to minimise both errors in data collection and missing item responses(de Leeuw, 2001). No imputation of missing values took place. Cleaning was limited to ‘sense checking’ data with any required corrections made and documented. Sixty-two completely blank rows (56 ACONF, 6 VOICE) were removed from survey responses where presumably a respondent had clicked the link but not consented or answered any questions. No responses were removed for being partially complete. Data from ALSPAC were screened by the ALSPAC study team for potentially identifying responses prior to being sent to the lead author (NH).

A measurement of deprivation was calculated using either Townsend (UK Data Service, 2020) (for participants in England) or Carstairs (Brown, Allik, Dundas, Leyland & H., 2014) (for participants in Scotland) scores from the respondent's postcode where provided. At the request of the ALSPAC study group, no postcode data was requested from Children of the 90 s. For partial postcodes, the most likely ward was assigned based on available digits of postcode. Both Townsend and Carstairs scores use a five-point scale to indicate deprivation with Townsend being the reverse of the Carstairs. Therefore, Townsend scores were transformed so that the Carstairs and Townsend scores matched. Deprivation is reported as a quintile (1–5), where 1 = most deprived, 5 = most affluent.

For the purposes of analysis, the respondents from VOICE, SAIL and SUPER were combined and referred to as PPI group participants.

Analysis consisted of summarising the demographics and survey responses which have been presented as numbers and percentages of respondents answering each question and the direction of response.

## Results

### Response Rate/Characteristics

A total of 1,684 completed surveys were received from 3 different groups of respondents (n = 1,226 from ALSPAC, 395 from ACONF, and 63 from PPI groups). The overall response rate for the survey was 16.4% with respondents taking an average of 12 min to respond. Response rates for individual respondent groups were as follows: ACONF (28.2%) and ALSPAC (20.9%), PPI groups (2.1%) comprised of: SAIL and SUPER groups (40%), Voice (1.7%). The low response from Voice was attributed to the fact that the questionnaire was promoted via the Voice weekly newsletter email once only although it remained accessible on the Voice website. A useable (full or partial) postcode was provided by 59% of respondents from ACONF and the PPI groups (17.6% of total respondents). A detailed summary of respondent characteristics is presented in Table 1.

**Table 1.** Respondent Characteristics.

Question	Response	Number of Respondents (%)							
		All respondents		ACONF		ALSPAC		PPI groups	
Study Source	Source	1,684	100	395	23.5	1,226	72.8	63	3.7
Q1 Have any of the following ever taken part in a research study?	You	264	58.7	218	56.3	n/a*	n/a	46	73.0
	Your child	6	1.3	6	1.6	n/a*	n/a	0	0
	You and your child	12	2.7	9	2.3	n/a*	n/a	3	4.8
	Neither	103	22.9	92	23.8	n/a*	n/a	11	17.5
	Not sure	65	14.4	62	16.0	n/a*	n/a	3	4.8
	Total	450	100	387	100	n/a*	n/a	63	100
Q2a Was your participation in the study as:	A person who had the health condition being studied	56	21.3	38	17.7	n/a*	n/a	18	37.5
	A healthy volunteer	161	61.2	135	62.8	n/a*	n/a	26	54.2
	A person who is at risk of developing the condition being studied	8	3.0	5	2.3	n/a*	n/a	3	6.3
	Not sure	38	14.5	37	17.2	n/a*	n/a	1	2.1
	Total	263	100	215	100	n/a*	n/a	48	100
Q2b Was your child's participation in the study as	A child who had the health condition being studied	4	25.0	3	23.1	n/a*	n/a	1	33.3
	A healthy volunteer	12	75.0	10	76.9	n/a*	n/a	2	66.7
	A person who is at risk of developing the condition being studied	0	0	0	0	n/a*	n/a	0	0
	Not sure	0	0	0	0	n/a*	n/a	0	0
	Total	16	100	13	100	n/a*	n/a	3	100
Q3a What was the experience of taking part in a study like for you	Very positive	639	42.9	66	30.8	550	44.9	23	46.9
	Positive	614	41.2	79	36.9	517	42.2	18	36.7
	Neither positive or negative	215	14.4	61	28.5	147	12.0	7	14.3
	Negative	5	0.3	2	0.9	<5**	0.1	1	2.0
	Very negative	0	0	0	0	<5**	0.1	0	0
	Not applicable	6	0.4	3	1.4	<5**	0.2	0	0
	Not sure	10	0.7	3	1.4	7	0.6	0	0
	Total	1,489	100	214	100	1,226	100	49	100
Q3b What was the experience of taking part in a study like for your child	Very positive	4	25.0	3	23.1	n/a*	n/a	1	33.3
	Positive	5	31.3	3	23.1	n/a*	n/a	2	66.7
	Neither positive or negative	4	25.0	4	30.8	n/a*	n/a	0	0
	Negative	0	0	0	0	n/a*	n/a	0	0
	Very negative	0	0	0	0	n/a*	n/a	0	0
	Not applicable	0	0	0	0	n/a*	n/a	0	0
	Not sure	3	18.8	3	23.1	n/a*	n/a	0	0
	Total	16	100	13	100	n/a*	n/a	3	100
Q23 Gender	Male	529	35.4	170	52.0	338	30.5	21	34.4
	Female	953	63.8	157	48.0	757	68.3	39	63.9
	Other	13	0.9	0	0	13	1.2	1	1.6
	Total	1,495	100	327	100	1,108	100	61	100
Q24 Age group	18–24	1	0.1	0	0	0	0	0	0
	25–44	1,116	74.7	0	0	1,109	100	7	12.1
	45–64	84	5.6	64	19.6	0	0	20	34.5
	65–74	283	19.0	262	80.4	0	0	21	36.2
	75–84	9	0.6	0	0	0	0	10	17.2
	85 and over	1	0.1	0	0	0	0	0	0
	Total	1,493	100	326	100	1,109	100	58	100
Q25 Ethnicity	White	1,445	97.0	322	99.4	1,066	96.2	57	98.3
	Mixed/multiple ethnic group	30	2.0	0	0	30	2.7	0	0
	Asian/Asian British	6	0.4	0	0	0	0	0	0
	Black/African/Caribbean/Black British	0	0	0	0	0	0	0	0
	Chinese	0	0	0	0	0	0	0	0
	Other Ethnic group	9	0.6**	2**	0.6	12 **	1.1	1**	1.7
	Total	1,490	100	324	100	1,108	100	58	100
Q26 Highest educational achievement	No qualifications	36	2.4	21	6.6	14	1.3	1	1.6
	O Levels/CSE/GCSE or equivalent	131	8.8	48	15.1	79	7.1	4	6.6
	AS/A Levels or equivalent	272	18.3	51	16.0	216	19.5	5	8.2
	Degree (e.g., BA, BSc) or equivalent	557	37.5	69	21.7	469	42.3	19	31.2
	Higher degree (e.g., MSc, PhD) or equivalent	229	15.4	27	8.5	186	16.8	16	26.2
	Professional qualifications (e.g., nursing, accountancy, teaching)	237	16.0	91	28.6	132	11.9	14	23.0
	Other	24	1.6	11	3.5	13	1.2	2	3.3
	Total	1,486	100	318	100	1,109	100	61	100
Q27 Overall health at the moment	Excellent	412	27.5	67	20.5	340	30.7	5	8.3
	Good	750	50.1	167	51.1	553	49.9	30	50.0
	Average	257	17.2	69	21.1	169	15.2	19	31.7
	Poor	58	3.9	19	5.8	36	3.3	3	5.0
	Very poor	13	0.9	1	0.3	9	0.8	3	5.0
	Not sure	6	0.4	4	1.2	<5**	0.2	0	0
	Total	1,496	100	327	100	1,109	100	60	100
Deprivation quintile	1	27	9.1	13	5.2	n/a*	n/a	14	31.1
	2	38	12.8	29	11.6	n/a*	n/a	9	20.0
	3	43	14.5	30	12.0	n/a*	n/a	13	28.9
	4	35	11.8	34	13.6	n/a*	n/a	1	2.2
	5	153	51.7	145	57.8	n/a*	n/a	8	17.8
	Total	296	100	251	100	n/a*	n/a	45	100

*ALSPAC were not asked about involvement in a study as it was known they were in a longitudinal study.

**Small numbers are grouped or disguised for tabulation purposes.

### Questions About Taking Part in Research

Generally, respondents found taking part in a research study (Q3a) a ‘positive’ (41.2%) or ‘very positive’ (42.9%) experience. Of respondents (non-ALSPAC) who were asked questions 1, 2a, 2b, and 3b, the majority (58.7%) reported that they themselves had taken part in a research study, and that they were a healthy volunteer (61.2%). Only 1.3% of non-ALSPAC respondents reported that their child had taken part in a study, 75% of whom were taking part as a healthy volunteer.

### RQ1 Attitudes Towards Sharing

The questionnaire survey measured respondents’ attitudes towards sharing through questions 5 to 11 and via example sharing scenarios. Respondents were asked how concerned they would be if they were informed that data from a study that they were involved in was being shared and the most common responses were ‘depends who it is shared with’ (29.7%) followed by ‘not very concerned’ (25.7%). PPI groups were more likely than ALSPAC or ACONF to be ‘not at all concerned’ with 35.5% of PPI respondents choosing that option compared to 25% (ACONF) and 14% (ALSPAC). Full results are displayed in Table 2.

**Table 2.** Question 5: How Concerned Would you be if you Knew Data from a Study That you Were Involved in was Being Shared?.

Response	Number of Respondents (%)							
	All respondents		ACONF		ALSPAC		PPI groups	
Very concerned	94	6.0	22	6.2	67	5.8	5	8.1
Somewhat concerned	309	19.7	45	12.6	258	22.5	6	9.7
Not very concerned	403	25.7	105	29.3	286	24.9	12	19.3
Not at all concerned	279	17.8	90	25.1	167	14.6	22	35.5
Not sure	17	1.1	6	1.7	11	1.0	0	0
Depends who it is shared with	465	29.7	90	25.1	358	31.2	17	27.4
Total	1,567	100	358	100	1,147	100	62	100

The majority of respondents were ‘not at all concerned’ about sharing with most of the suggested organisations, although slightly higher levels of concern were observed for sharing with a pharmaceutical company (∼21% ‘concerned’ as compared to <10% for universities or hospitals). More concern was exhibited for sharing with the government with 30.2% of respondents ‘somewhat concerned’ and 18.6% ‘very concerned’ as compared to sharing with organisations such as universities, hospitals, or charities. Unsurprisingly, a spike in concern was observed for sharing data ‘on the internet for anyone to use’ with 61% of respondents ‘very concerned’ about this (Table 3).

**Table 3.** Question 6 how Concerned Would you be if you Knew Data was Being Shared with.

	Response	Number of Respondents (%)							
		All respondents		ACONF		ALSPAC		PPI groups	
Researchers at the same organisation where your data was collected	Very concerned	12	0.8	3	0.8	8	0.7	1	1.6
	Somewhat concerned	45	2.9	11	3.1	27	2.3	7	11.5
	Not very concerned	408	25.0	119	33.3	269	23.4	20	32.8
	Not at all concerned	1,094	69.7	219	61.3	842	73.1	33	54.1
	Not sure	11	0.7	5	1.4	6	0.5	0	0
	Total	1,570	100	357	100	1,152	100	61	100
Researchers at a pharmaceutical company, e.g., for developing new medicines	Very concerned	73	4.7	19	5.4	46	4.0	8	12.9
	Somewhat concerned	258	16.5	53	15.0	189	16.4	16	25.8
	Not very concerned	557	35.6	129	36.4	410	35.7	18	29.0
	Not at all concerned	652	41.6	146	41.2	487	42.4	19	30.7
	Not sure	26	1.7	7	2.0	18	1.6	1	1.6
	Total	1,566	100	354	100	1,150	100	62	100
Researchers at a university	Very concerned	24	1.5	7	2.0	14	1.2	3	4.9
	Somewhat concerned	116	7.4	27	7.6	79	6.9	10	16.4
	Not very concerned	582	37.1	141	39.7	427	37.1	14	23.0
	Not at all concerned	829	52.9	177	49.9	619	53.7	33	54.1
	Not sure	17	1.1	3	0.9	13	1.1	1	1.6
	Total	1,568	100	355	100	1,149	100	61	100
Researchers at a hospital	Very concerned	23	1.5	6	1.7	15	1.3	2	3.2
	Somewhat concerned	87	5.6	17	4.8	61	5.3	9	14.5
	Not very concerned	543	34.7	131	37.2	395	34.4	17	27.4
	Not at all concerned	899	57.5	196	55.7	670	58.3	33	53.2
	Not sure	11	0.7	2	0.6	8	0.7	1	1.6
	Total	1,563	100	352	100	1,149	100	62	100
Researchers in another country	Very concerned	193	12.3	61	17.0	121	10.5	11	17.7
	Somewhat concerned	405	25.8	92	25.7	297	25.9	16	25.8
	Not very concerned	441	28.1	97	27.1	328	28.6	16	25.8
	Not at all concerned	473	30.2	94	26.3	364	31.7	15	24.2
	Not sure	56	3.6	14	3.9	38	3.3	4	6.5
	Total	1,568	100	358	100	1,148	100	62	100
A charity or not for profit organisation	Very concerned	106	6.8	44	12.4	58	5.0	4	6.5
	Somewhat concerned	349	22.3	73	20.5	259	22.5	17	27.4
	Not very concerned	521	33.2	120	33.7	391	34.0	10	16.1
	Not at all concerned	539	34.4	99	27.8	413	35.9	27	43.6
	Not sure	53	3.4	20	5.6	29	2.5	4	6.5
	Total	1,568	100	356	100	1,150	100	62	100
The government	Very concerned	292	18.6	77	21.5	199	17.3	16	25.8
	Somewhat concerned	474	30.2	94	26.3	366	31.8	14	22.6
	Not very concerned	423	26.9	102	28.5	307	26.7	14	22.6
	Not at all concerned	326	20.8	66	18.4	243	21.1	17	27.4
	Not sure	55	3.5	19	5.3	35	3.0	1	1.6
	Total	1,570	100	358	100	1,150	100	62	100
A student at a university	Very concerned	217	13.8	54	15.1	150	13.1	13	21.0
	Somewhat concerned	388	24.7	70	19.6	305	26.5	13	21.0
	Not very concerned	497	31.7	137	38.4	346	30.1	14	22.6
	Not at all concerned	425	27.1	86	2.8	323	28.1	16	25.8
	Not sure	41	2.6	10	2.8	25	2.2	6	9.7
	Total	1,568	100	357	100	1,149	100	62	100
On the internet for anyone to use	Very concerned	956	61.0	240	67.6	681	59.2	35	56.5
	Somewhat concerned	367	23.4	63	17.8	286	24.9	18	29.0
	Not very concerned	111	7.1	20	5.6	89	7.7	2	3.2
	Not at all concerned	95	6.1	21	5.9	70	6.1	4	6.5
	Not sure	38	2.4	11	3.1	24	2.1	3	4.8
	Total	1,567	100	355	100	1,150	100	62	100

Respondents’ main concerns regarding sharing (Table 4) were harms relating to the security of their data; being identified (64.7%) or having their data stolen (68.6%). Respondents were more likely to be ‘very concerned’ about embarrassment if their data were linked back to them (54.6%) than about data being used for profit (44.2%), data being misinterpreted (44.3%), or researcher-related issues such as lack of acknowledgement of the original research team (39.3%). Respondents were ‘most concerned’ about being identified in the data (46.1%) (Supplementary material Q7a).

**Table 4.** Question 7 If Data from the Study in Which you Were Involved was Being Shared, how Concerned Would you be About the Following?.

	Response	Number of Respondents (%)							
		All respondents		ACONF		ALSPAC		PPI groups	
a) If I could still be identified in the data	Very concerned	1,009	64.7	203	51.4	763	66.4	43	70.5
	Somewhat concerned	408	26.2	112	28.4	286	24.9	10	16.4
	Not very concerned	96	6.2	1	6.6	65	5.7	5	8.2
	Not at all concerned	35	2.2	7	1.8	25	2.2	3	4.9
	Not sure	12	0.8	26	6.6	11	1.0	0	0
	Total	1,560	100	395	100	1,150	100	61	100
b) If my data could be used in research I don’t approve of	Very concerned	664	42.7	187	47.3	438	38.2	39	62.9
	Somewhat concerned	554	35.6	103	26.1	438	38.2	13	21.0
	Not very concerned	195	12.5	9	2.3	154	13.4	7	11.3
	Not at all concerned	116	7.5	14	3.5	99	8.6	3	4.8
	Not sure	28	1.8	34	8.6	19	1.7	0	0
	Total	1,557	100	395	100	1,148	100	62	100
c) If my data could be stolen	Very concerned	1,068	68.6	282	81.5	742	64.6	44	71.0
	Somewhat concerned	356	22.9	46	13.3	296	25.8	14	22.6
	Not very concerned	90	5.8	2	0.6	74	6.4	4	6.5
	Not at all concerned	36	2.3	4	1.2	32	2.8	0	0
	Not sure	7	0.5	12	3.5	5	0.4	0	0
	Total	1,557	100	346	100	1,149	100	62	100
d) If my data could be used for making a profit e.g., advertising instead of research	Very concerned	689	44.2	0	0	689	60.0	0	0
	Somewhat concerned	631	40.5	267	77.0	322	28.0	42	67.7
	Not very concerned	172	11.0	60	17.3	96	8.4	16	25.8
	Not at all concerned	37	2.4	4	1.2	33	2.9	0	0
	Not sure	29	1.9	16	4.6	9	0.8	4	6.5
	Total	1,558	100	347	100	1,149	100	62	100
e) If it would be embarrassing if my data was linked back to me	Very concerned	850	54.6	212	61.1	597	52.0	41	66.1
	Somewhat concerned	409	26.3	95	27.4	303	26.4	11	17.7
	Not very concerned	179	11.5	23	6.6	148	12.9	8	12.9
	Not at all concerned	100	6.4	11	3.2	87	7.6	2	3.2
	Not sure	20	1.3	6	1.7	14	1.2	0	0
	Total	1,558	100	347	100	1,149	100	62	100
f) If people could misinterpret the data and come to the wrong conclusions	Very concerned	691	44.3	215	62.0	433	37.6	43	69.4
	Somewhat concerned	541	24.7	109	31.4	418	36.3	14	22.6
	Not very concerned	221	14.2	15	4.3	203	17.6	3	4.8
	Not at all concerned	81	5.2	3	0.9	76	6.6	2	3.2
	Not sure	26	1.7	5	1.4	21	1.8	0	0
	Total	1,560	100	347	100	1,151	100	62	100
g) If the original research team didn’t get credit for collecting the data	Very concerned	612	39.3	157	45.2	429	37.3	26	41.9
	Somewhat concerned	596	38.3	145	41.8	424	36.9	27	43.6
	Not very concerned	212	13.6	33	9.5	175	15.2	4	6.5
	Not at all concerned	102	6.6	8	2.3	90	7.8	4	6.5
	Not sure	36	2.3	4	1.2	31	2.7	0	0
	Total	1,558	100	347	100	1,149	100	62	100
h) If it stopped researchers doing their own original research	Very concerned	583	37.4	162	46.6	389	33.8	32	51.6
	Somewhat concerned	521	33.4	139	39.9	357	31.0	25	40.3
	Not very concerned	279	17.9	35	10.1	242	21.0	2	3.2
	Not at all concerned	108	6.9	7	2.0	100	8.7	1	1.6
	Not sure	70	4.5	5	1.4	63	5.5	2	3.2
	Total	1,561	100	348	100	1,151	100	62	100

When asked about the likelihood of granting permission for their data to be used for various purposes (Supplementary Material Q8), the majority of respondents indicated that they were ‘very likely’ to (agree to) share for research by a university (62.6%), a hospital (72.2%) or to inform the public about a health issue (47.8%). The majority of respondents were only ‘somewhat’ sure about agreeing to share with pharmaceutical companies (39.6%), to help students get data for a project (37.3%) and ‘to help the government study health problems’ (39.1%). PPI groups were less likely to agree and more likely to object to sharing their data ‘To help a pharmaceutical company do research’ than ALSPAC or ACONF with 23% of PPI respondents selecting that they would be ‘very likely’ to allow this and 8.2% of them selecting that they would be ‘very unlikely’ to allow this as compared to approximately 36% and approximately 3% respectively selected by ALSPAC, ACONF and respondents overall. Conversely, PPI groups seemed slightly more relaxed about sharing data for student projects than the other respondents, with 42.6% of them saying that they would be ‘very likely’ to share for this purpose as compared to approximately 30% selected by ALSPAC, ACONF and respondents overall.

All statements of potential benefit were popular (Supplementary Material Q9), but the aspect of sharing that respondents seemed to find most beneficial was: ‘Rarer diseases and conditions can be studied more easily using combined data, without having to wait for more studies’ (81.6%). Similarly, all potential motivations to share were popular (Supplementary Material Q10), but privacy (assured anonymity) and altruism (chance to help others) were joint most important with approximately 93% of respondents selecting these statements. ACONF and PPI groups were more likely than the other respondents to select that a chance to understand their own condition better would motivate them, with 78.3% of PPI members and 80.6% of ACONF respondents selecting this statement as compared to ALSPAC respondents (64.3%).

Respondents were asked which details about themselves (such as age, employment, alcohol use) they would be willing to share in an anonymised data set (Supplementary Material Q11). The majority of respondents (45–58%) were ‘very willing’ to share all fifteen suggested details. Details of mental health (45.6%) and employment (45.4%) had the fewest respondents who were ‘very willing’ to share, but only marginally as compared to family history of disease (47.9%) for example. Few respondents were ‘not at all willing’ to share any of the fifteen types of data.

### RQ2 Does Knowing About Sharing Affect Taking Part

When respondents were asked whether knowing that their data would be shared would affect their decision to take part in a study, 47.2% responded that this would have no effect on their taking part (Table 5). Thirty nine percent would be ‘a bit more cautious about taking part’. Of respondents who would be a bit more cautious, ALSPAC respondents appeared to be more likely to be cautious than other respondents with 40.8% selecting this statement as compared to nearer 30% of cautious ACONF and PPI respondents. Very few respondents stated that they would be much more or less likely to take part after learning about sharing (3–4%) and even fewer (0.8%) reported that they would not take part at all.

**Table 5.** Question 15: if you Knew Your Data Might be Shared, What Affect Would it Have on you Taking Part in a Study?.

Response	Number of Respondents (%)							
	All respondents		ACONF		ALSPAC		PPI groups	
I would not take part at all	12	0.8	1	0.3	9	0.8	2	3.3
I’d be much less likely to take part	54	3.6	19	5.7	32	2.9	3	4.9
I’d be a bit more cautious about taking part	586	38.7	111	33.3	456	40.8	19	31.2
It would have no effect on my decision	714	47.2	172	51.7	516	46.1	26	42.6
I’d be a bit more likely to take part	35	2.3	12	3.6	21	1.9	2	3.3
I’d be much more likely to take part	47	3.1	12	3.6	28	2.5	7	11.5
Not sure	65	4.3	6	1.8	57	5.1	2	3.3
Total	1,513	100	333	100	1,119	100	61	100

### RQ3 Preferences for Sharing

When asked about their consent preferences (Table 6), respondents were almost evenly split between agreeing that a single consent at the beginning of the original study could cover all future sharing (39%) and wanting to re-consent each time the data is shared with the option to say no (41.7%). Results by cohort reveal that only ALSPAC respondents exhibited a preference for consent each time data was shared (43.9%). Just 5.4% of respondents stated that they were happy for their data to be shared without being consulted at all.

**Table 6.** Question 12: how and When Would you Like to be Asked to Share Your Data?.

Response	Number of Respondents (%)							
	All respondents		ACONF		ALSPAC		PPI groups	
Once, on the consent form for the original study	589	39.0	125	37.5	434	38.9	30	49.2
Every time it is shared, with the option for me to say no	631	41.7	119	35.7	490	43.9	22	36.1
Just let me know every time it is shared	135	8.9	29	8.7	104	9.3	2	3.3
There is no need to ask me, just share it	82	5.4	34	10.2	44	3.9	4	6.6
I have no preference	73	4.8	26	7.8	44	3.9	3	4.9
Total	1,510	100	333	100	1,116	100	61	100

Almost all respondents (97.4%) agreed that the consent form should explain that their data could be shared (Supplementary Material Q13), and approximately 90% of respondents thought that explanations should be provided on how the researchers would protect respondents’ identities, who might benefit from using their data and with whom the data might be shared. Respondents were less interested in how or where the data would be stored. Only 1% thought that none of these things would convince them to share their data. When asked how important it was that they were informed in the consent form that their data might be shared, the majority of respondents (66.3%) thought it ‘very important’. PPI groups were more likely to find it ‘very important’ than ACONF or ALSPAC with 78.7% of them selecting this response compared to 64.9% and 66% respectively (Table 7).

**Table 7.** Question 14: how Important is it That you are Informed on the Consent Form That Your Study Data Might be Shared?.

Response	Number of Respondents (%)							
	All respondents		ACONF		ALSPAC		PPI groups	
Very important	1,002	66.3	216	64.9	738	66.0	48	78.7
Somewhat important	399	26.4	84	25.2	308	27.6	7	11.5
Not very important	79	5.2	24	7.2	51	4.6	4	6.6
Not at all important	24	1.6	7	2.1	15	1.3	2	3.3
Not sure	8	0.5	2	0.6	6	0.5	0	0
Total	1,512	100	333	100	1,118	100	61	100

When given the chance to state whether they would prefer to give consent separately for each type of organisation that data could be shared with most respondents (60.1%) answered ‘yes’ (Supplementary Material Q16).

Respondents were also asked about whether they thought a register of participants who were willing to share their data was a good idea (Supplementary Material Q17). Most (63.8%) thought that this was a good idea, but fewer (50.7%) would agree to be named on such a register (Supplementary Material Q18).

Respondents were given a brief statement about storage of data with controlled or open access and then asked how they would prefer their study data to be stored (Table 8). The majority preferred controlled access (86.9%) with only 3.7% preferring open access (Table 8).

**Table 8.** Question 19: how Would you Prefer Your Study Data to be Stored?.

Response	No. of Respondents (%)							
	All respondents		ACONF		ALSPAC		PPI groups	
Open access	56	3.7	16	4.9	35	3.2	5	8.2
Controlled access	1,301	86.9	289	88.4	958	86.3	54	88.5
No preference	76	5.1	17	5.2	58	5.2	1	1.6
Not sure	65	4.3	5	1.5	59	5.3	1	1.6
Total	1,498	100	327	100	1,110	100	61	100

Respondents were then asked who they thought should give permission to share data in a controlled access model (Supplementary Material Q20). The majority of respondents (38.8%) thought that the participants who took part in the study should decide, followed by the organisation where the data was collected (23.3%). The option for an independent committee was more likely to be selected by PPI groups (15.3%) compared to ALSPAC and ACONF (9%).

When questioned about data ownership (Supplementary Material Q21) the majority of respondents (49.4%) selected ‘me/the participants who took part’ followed by ‘the researcher(s) who collected it’ (45.2%) and ‘the organisation where the original researcher(s) work’ (44.1%) as the owners of data. Only 1.5% (n = 26) thought that ‘anyone who uses it’ should own the data.

Finally, the questionnaire asked respondents whether they thought that feedback on how their data were used in secondary research was important, with ∼82% of respondents indicating that this was indeed ‘important’ to them (Supplementary Material).

10% of respondents left a free text comment about sharing as categorised in Supplementary Material Q29. The majority of comments remarked about the ideal type of secondary use or recipients. Second to this were comments about being happy for their data to be shared for secondary use with the caveat that they must not be identifiable. A number of respondents commented on how their views or experience influenced the way in which they answered the questions.

## Discussion

The responses to this questionnaire survey demonstrated that respondents of longitudinal studies and members of PPI groups are generally open to data sharing and exhibit low levels of concern when asked about sharing most types of data. Nevertheless, just because participants are generally happy to share, it should not be assumed that researchers should do so without asking. The survey also provided evidence of participants’ concerns about potential sharing-related harms. When prompted, they were able to state their preferences for sharing procedures such as consent and storage.

Respondents’ concerns regarding sharing depended on with whom the data were to be shared; this reflects previous research which showed that participants are happy to share data but with caveats about who could use it (Baines et al., 2024; Hamilton et al., 2024; Mello et al., 2018; Mozersky et al., 2020; Nagappan & Zhu, 2025; Shah et al., 2018). For example, it has previously been reported that participants are more likely to agree to share with university scientists or “*qualified researchers”* chosen because of their credentials to keep their data in the “*research eco-system”* where possible (K. P. Manhas et al., 2015; Mozersky et al., 2020 p. 2205) and are least likely to want to share with drug companies, other commercial enterprises, insurance companies, industry researchers, for-profit organisations and third parties in general (Biasiotto et al., 2023; Dotter et al., 2025; Kerasidou & Kerasidou, 2023; Mello et al., 2018; Mozersky et al., 2020; Shah et al., 2018; Tosoni et al., 2022). Participants have referred to “*slippery slopes”* where data are shared further and further from originally agreed projects as time goes on (Mhairi Aitken et al., 2016 p. 16) and differential “*legal and ethical norms”* in commercial enterprises (Tosoni et al., 2022 p. 12). The importance of with whom data were shared was also reiterated in the free text comments from questionnaire respondents in the current study.

The survey was administered prior to and during the early months of the COVID-19 pandemic, a period that intensified public discourse on trust in government and science as well as use and access to data which played a critical role in outbreak management. Literature emerging from the COVID-19 pandemic exploring privacy, security and trust indicates that public trust in governments, science and health organisations was generally high, which may have affected survey respondents’ willingness to share data for health research (Enria et al., 2021; Sibley et al., 2020; Summers et al., 2022). Comparable patterns have been observed in studies of public acceptability of data-sharing for technologies such as contact-tracing applications, where uncertainty and perceived health risks brought about by the pandemic heightened support for technological solutions, often overriding privacy concerns (Freddi & Wasenden, 2024; Siegrist & Bearth, 2021; Williams et al., 2021; Wnuk et al., 2020). Consistent with our findings, studies examining data sharing report maintenance of broad but conditional or even increased support for sharing personal health data with secondary or third parties during the pandemic (Health Data Research UK, 2025; Jones et al., 2022; Summers et al., 2022). The exact influence of the pandemic on our respondents’ attitudes towards data sharing remains uncertain. Although respondents may have been willing to share (anonymised) health data for largely altruistic reasons, had the survey been conducted later in the pandemic, attitudes towards trust privacy, and willingness to share may have shifted, particularly with respect of organisations they trusted least.

Pertinent to university research, just over a third of questionnaire respondents were happy to share their data for student projects. There is sparse evidence about participants’ attitudes to student access to data, with mixed responses (Hate et al., 2015; Mozersky et al., 2020). Some participants would be happy for students to have access to data for training in analysis techniques (Mozersky et al., 2020) whilst others were divided with some happy to share with students and others suggesting that students should make the effort to collect primary data for their own education (Hate et al., 2015).

On the other hand, it is also possible that had respondents to this survey not been provided with the ‘depends who…’ response option, they would have instead selected ‘not very concerned’ which was the second most frequently endorsed category. Generally, respondents being ‘not very concerned’ about sharing tallies with findings from previous research where levels of concern were low, but specific harms were identified when prompted (Clerkin et al., 2013; Courbier et al., 2019; National Academies of Sciences & Medicine, 2020). Some 61% of respondents in the current study were ‘very concerned’ regarding the potential for sharing data ‘on the internet for anyone to use’ (by which we can infer a completely open access model). We cannot determine whether this is due to privacy concerns (although respondents were told that data would be anonymised before any sharing took place) or because respondents want to know who will be using their data. In addition, the degree to which survey respondents (and participants in general) understand anonymisation is unknown.

Prime concerns relative to privacy; being identified or having their data stolen echo previous research which identified the most commonly raised concerns to be re-identification, ‘misuse’ of data (including theft, misinterpretation and use for purposes not aligned with participant values) as well as use of data for profit (Baines et al., 2024; Cascini et al., 2024; Colombo et al., 2019; Hamilton et al., 2024; Mello et al., 2018; Mozersky et al., 2020; Naude et al., 2025; Sánchez et al., 2023; Stockdale, Cassell & Ford, 2018; Watson et al., 2023). Our survey did not elicit whether respondents made the distinction between being directly identifiable or whether identification could occur through a combination of variables.

Some respondents exhibited concern for researcher-centric issues such as lack of recognition for the original research team, misinterpretation of data and the potential for the act of sharing stopping researchers from doing their own original research. Previous research has also reported participants’ identification of potential harms that were researcher orientated, such as data misinterpretation biasing secondary results (Mozersky et al., 2020), or “*poor quality science”* being conducted by secondary researchers (Mello et al., 2018 p. 2206). Such findings are, however, largely from qualitative research, where participants were encouraged to discuss such issues. It is possible that these harms were selected simply because the options were provided, and they are not key or spontaneous concerns for questionnaire respondents. However, by discussing and emphasising potential researcher-related issues, participants demonstrate that they would prefer that their data are used well, maximising the benefits of sharing (Carr & Littler, 2015; Loder & Groves, 2015; Mello et al., 2013). There is no point in participants risking their health, giving up their time or allowing their data to be shared if it is not used appropriately.

Similar to our questionnaire respondents, previous research has also reported an expectation of data sharing providing benefits to the general public, including specifically to the communities who contributed the data (Cervera de la Cruz & Shabani, 2023; Mello et al., 2018; Watson et al., 2023). It has also been demonstrated that, similar to our questionnaire respondents, research participants appreciate the benefits of sharing to science and discovery (Colombo et al., 2019; Jao et al., 2015; Mozersky et al., 2020). During consent, researchers should explain the benefits of sharing to the participants themselves, including reducing participant burden and maximisation of participant contributions as well as the usual and oft-cited benefits to science and research.

Respondents’ willingness to share all fifteen potential anonymised details about themselves with a maximum range of just 10% between data types could mean that respondents were not fully engaged with these questions. On reflection a more concise list of data types could have been presented. Alternatively, respondents may have been genuinely unconcerned, understanding the explanation that the hypothetical data would be anonymised. If we are to make any inference from the current data, it could be participants were less willing to have mental health and employment data shared. In this aspect, the questionnaire respondents were perhaps more conservative than those in existing literature (Cheah et al., 2018; Shah et al., 2018). Some participants in previous literature even thought that they would not mind if the data were *not* anonymised if it was something like a diagnosis but would want data anonymised if it referred to sexual activity or alcohol consumption (Mozersky et al., 2020). Participants may need clear reminders about anonymisation so that they understand what they disclose is very unlikely to be attributable to themselves. Researchers should be mindful that participants may in fact distinguish between sensitive and non-sensitive data (Baines et al., 2024; Sánchez et al., 2023; Watson et al., 2023) and consider whether seemingly innocuous identifiers such as employment or occupation need to be shared or even collected (Corti et al., 2014; Lowrance, 2002; Tudur Smith et al., 2015; UK Data Service, 2016).

Little supporting evidence exists regarding whether or not knowing about sharing would discourage participants from taking part in research. Indeed, previous studies have demonstrated mixed degrees of understanding of data sharing in the first place, with some participants being unaware whether or not their data might already have been shared (Baines et al., 2024; Manhas et al., 2016; Mursaleen et al., 2017b). Generally, existing evidence has demonstrated that once participants have been informed about data sharing, they want more information about it, including with whom their data might be shared, but overall researchers are trusted to make sharing decisions on participants’ behalf (Manhas et al., 2015; Mozersky et al., 2020).

Respondents thought it ‘very important’ that the original study consent form lets participants know that their data will be shared, giving an explanation about sharing, how their identity would be protected via anonymisation, with whom the data might be shared and who might benefit. This aligns with existing evidence indicating that the participant information sheet and consent form could play an educational role in explaining data sharing to participants (Manhas et al., 2018; Mozersky et al., 2020). A fully informed consent describing what research may be conducted and by whom, privacy and security measures and how studies could benefit participants has been linked both to trust and to a sense of control (Mhairi Aitken et al., 2016; Baines et al., 2024; Broekstra et al., 2020; Cervera de la Cruz & Shabani, 2023; Kalkman et al., 2019; Milne et al., 2021). However, trust in institutions alone does not mean that participants are happy to consent to researchers sharing their data with just anyone; instead trust is linked to, and must be combined with transparency about security and future uses (Mhairi Aitken et al., 2016; Naude et al., 2025; Waind, 2020). If participants do not understand exactly what data sharing involves, this could be considered “*an unsafe consent”* (Mursaleen et al., 2017b p. 527).

Consistent with some research (Chan et al., 2012; Clerkin et al., 2013; Taylor & Taylor, 2014; Wild et al., 2023), overall questionnaire respondents exhibited a slight preference for re-consent each time their data are shared. Conversely, other evidence suggests that participants would prefer to engage little with researchers once the original research was over, avoiding the burden of re-contact for re-consent each time their data was requested for sharing (Cheah et al., 2018; Köngeter et al., 2022; Manhas et al., 2016; Manhas et al., 2018; Mello et al., 2018). This was the second most popular option for questionnaire respondents. However, if we look at questionnaire responses from individual studies, only ALSPAC respondents exhibited a preference for consent each time data was shared. It is not clear whether this is due to the age of the cohort, the contact model established with the ALSPAC study coordinators whereby participants can opt-out of research or linkage or something else. Just 4.9% of all respondents stated that they were happy for their data to be shared without being consulted at all highlighting the importance of a thorough consent process.

Respondents gave a strong indication that their data should be stored with controlled access. Some contrary evidence exists for example where 39% of participants thought that access should be “*broad”*, open not only to researchers, but also “*other groups and individuals such as patients’ and citizen group representatives and journalists”* (Colombo et al., 2019 p. 5). However, broad access does not necessarily equate to open access; controlled access according to the FAIR principles (Wilkinson et al., 2016) can still ensure that data is used widely for secondary research. Where data are stored will impact its accessibility and also the length of time for which it is available. Repository choices will need to honour participants’ consent. If researchers do decide to seek consent for complete open access, they should be clear that open access can neither preclude any future users such as commercial organisations nor make promises that data will only be used for research purposes (Attwood & Munafò, 2016).

Aligning with their views on re-consent, if data were made available via controlled access, respondents favoured permission for access being given by the participants who took part in the study rather than by ‘the organisation where the data was collected’. Previous research has emphasised governance issues with many references to the role of accountable committees (with lay or participant members) or gatekeepers who could make decisions on access or sharing requests (Cheah et al., 2018; Colombo et al., 2019; Köngeter et al., 2022; Manhas et al., 2016; Manhas et al., 2018; Shah et al., 2018). More recently, movements towards participatory data governance and CARE principles in combination with FAIR principles (Carroll et al., 2021) can ensure the interests of participants are protected throughout the lifespan of datasets (Carroll et al., 2021; Chukwurah et al., 2024). By contrast, few questionnaire respondents chose a committee as their preferred option for making sharing decisions. It should be noted, however, that the majority of questionnaire respondents in the current study were from ALSPAC or ACONF and were therefore familiar with re-contact by their original study team regarding further research; and perhaps envisaged that all research participants could be contacted in this way. Participants in previous research reported that they trusted the original researchers to make sharing decisions on their behalf (K. P. Manhas et al., 2015; Kiran Pohar Manhas et al., 2016; Mello et al., 2018; Mozersky et al., 2020). However, researchers cannot bank on the trust that participants hold in the original research team to gain consent if they know that ultimately requests will be handled elsewhere (Mhairi Aitken et al., 2016; Mello et al., 2018; Mozersky et al., 2020).

Interestingly when asked about ownership, the greatest number of questionnaire respondents thought that ‘the participants who took part’ owned their research data. The question in the survey intended to gather views on ownership of *anonymised* research data, but as with other survey questions it is not clear whether respondents were thinking of anonymised datasets or personal data (over which participants do arguably have greater control). Depending on funding arrangements, jurisdictions and disciplines, it is highly unlikely that participants are ever considered owners of their own data, and often the researchers themselves are merely data custodians; research data are usually owned by funders, with a whole host of other potential candidates and stakeholders (researchers, host institutions, sponsors or journals) (Cleary et al., 2013). Confusion about who owns data can also be observed in the literature (Mursaleen et al., 2017a; Mursaleen et al., 2017b; Wild et al., 2023). The distinction between ownership, custodianship, use with consent and use without consent for the public benefit and legal use versus ethical use all needs to be unpicked and explained to participants as part of the consent process.

Finally, some existing evidence on feedback to participants suggests limiting communications to updates on projects in which individuals actually took part or just sending notifications when their data is used for secondary research (Manhas et al., 2018; Mursaleen et al., 2017b). Engaging participants by keeping them informed of the types of studies that are using their data, and the resultant outcomes enforces researcher accountability, but may also increase transparency and trust (Aitken et al., 2016a; Cervera de la Cruz & Shabani, 2023; Milne et al., 2019; Nwebonyi et al., 2022; The Patients Association, 2023; Tosoni et al., 2022; Yusuf et al., 2024). Positive messages about how data is used, lets participants know that research is benefitting them and others (Aitken et al., 2016a; Damschroder et al., 2007). Informing participants of expected communications, and how researchers will maintain up-to-date contact details at the outset prevents them feeling burdened by further contact and gives the opportunity for opt out.

Overall, few differences in terms of attitudes towards sharing or preferences for sharing procedures were exhibited between the included cohorts. However, it is notable that PPI groups were more likely to be ‘not at all concerned’ about their data being shared, and were more open to sharing with students, but more likely to object to sharing their data with pharmaceutical companies for research purposes. PPI groups also appeared to be slightly more in favour of committees for the purposes of sharing decisions. In some respects, ALSPAC gave more conservative answers, being more likely to be cautious about taking part in a study where their data would be shared, and exhibiting a preference for individual consent each time it was shared. It is not clear whether these differences are attributable to the age or experiences of respondents or influenced by being part of a longitudinal study (or not) or a combination of all these factors. Some research has established that older age is associated with increased support for sharing (Kalkman et al., 2019) whilst a review has identified mixed results (Hutchings et al., 2020). Factors such as levels of trust in researchers, levels of concern about privacy, education and employment were also important (Hutchings et al., 2020). ALSPAC respondents may be used to established contact and consent processes between study coordinators (e.g., being given the option to opt in and out of research regularly and knowing that secondary studies are screened) and therefore reflected these processes in their responses. ACONF and PPI groups were more likely to be persuaded to share by having the chance to understand their own condition better, perhaps attributable to the older ages of both of these cohorts (where health conditions may be more common).

### Strengths and Limitations

This questionnaire survey achieved a relatively large sample of 1,664 respondents; larger than some other published surveys on attitudes to data sharing (Colombo et al., 2019; Ludman et al., 2010; Manhas et al., 2018; Mello et al., 2018; Mursaleen et al., 2017b; Shah et al., 2018; Willison et al., 2009).

The participants of the two longitudinal studies from which survey respondents were sampled ranged in age from their late-20 s to their 70 s and were from opposite ends of the UK. By virtue of being a birth cohort (ALSPAC) and a cross-sectional study based on year of birth (ACONF), these two groups can claim to be broadly representative of the general population, at least of the localities from which the cohorts were assembled. Respondents from both groups were representative of their respective study populations. While there are many such regional and national longitudinal cohorts, with some dating back to the 1940s (Pearce et al., 2009) there is surprisingly very little published literature (with the exception of some studies on data linkage (Aitken et al., 2016b; Skatova et al., 2019) which examines the attitudes of longitudinal study participants to data sharing for secondary research and so the results of this study provide valuable corroborative evidence for researchers working with longitudinal cohorts.

However, a significant limitation is the inherent bias both in respect to the sample of respondents selected (sampling bias) and the responses to the questionnaire (response bias and non-response bias) affecting generalisability of results. The questionnaire was long, and some, though not many, respondents seemed to get fatigued towards the end of the questionnaire with slight drop out. The survey was conducted a number of years prior to publication, and attitudes may since have changed. A final caveat of this is that this work only managed to achieve participants who took part in longitudinal studies. Ironically, lack of robust data sharing protocols, with consent in place for re-contact, precluded inclusion of participants in clinical trials. We also had no way of knowing what other type of studies (if any) PPI group participants took part in and, (although the survey gave guidance), which type of data, research or implications participants were considering when answering the survey questions.

## Best Practices

Brief recommendations for researchers are positioned throughout this article and are summarised below. It is not anticipated that these recommendations will require a great degree of policy change at a high level (e.g., from funders or journals), instead they comprise practical steps that researchers could take to align their research with the preferences of participants whilst still operating within the overarching guidance of research funders.

To provide participants with enough information for fully informed consent, researchers should provide an introductory statement of what sharing actually is, who might benefit, exactly which data will be shared and how data will be anonymised and stored securely prior to sharing.

Consent forms should include a simple statement regarding with whom data might be shared and for what purpose, selected for example from broad categories such as researchers (or students) in other institutions such as other universities, hospitals or commercial organisations and ‘further research’ or ‘to combine with other datasets for analysis’.

Researchers should also state who will be making decisions on sharing on behalf of participants, and where possible include participants in decision making.

Study data should be stored with controlled access, ensuring that data is not released to secondary researchers without following a request procedure. In line with respondent preferences, data should not be freely available (‘on the internet for anyone’ or in a repository with no access permission required).

Guidance and researchers should stop suggesting re-consent as an option. One-off, properly informed consent at the point of joining the original research study reduces the need for burdensome re-consent processes that might be beyond the capacity of already stretched researchers as well as being intrusive for participants.

Researchers should consider: who is the moral owner of the data? “*Is this data set mine, or does it really belong to the patients in the trial, and do I act merely as custodian?”* (Vickers, 2006). If researchers believe that the participants own the data, they may manage it and use it in the way that participants want.

Briefly, in the spirit of transparency, researchers need to commit to providing feedback on use of participants’ data for secondary research.

## Research Agenda

Although the questionnaire was useful in determining the majority opinion, it could not explain the reasons behind these opinions, which could be further explored with qualitative methods. Some of the data gathered in the questionnaire needs verifying with further research, where there is not already a large volume of evidence in existing literature for example: whether or not knowing about sharing would discourage participants from taking part in research; preferred repository types; attitudes towards sharing data with students; and how participants view ownership of shared data. Organisations may wish to consider development of informative statements for information sheets or consent forms, and testing the relevance and acceptance of their own data sharing polices with the participants they concern or through co-production.

## Educational Implications

The study clearly identifies some longitudinal study and PPI group participants’ preferences for data sharing including consent and data storage. Some of these findings align with existing evidence, underlining the need for a participant focussed consent, and planning for sharing from the outset of studies to ensure that data management and sharing is reflective both of consent and of participant preferences. Other findings contrast with existing evidence, for example the desire for individual consent. We need to ensure that we do not treat all participant groups as homogenous; perhaps there are differences in sharing or consent requirements between participants taking part in different types of studies or providing different types of data. Some of this evidence would benefit from further exploration; such as perceptions of data ownership. Researchers should use this evidence in their own practice to ensure that consent for sharing is always fully informed and that participant data is treated in alignment with participant values and preferences.

## Supplemental Material

sj-docx-1-jre-10.1177_15562646261452724 - Supplemental material for Attitudes of Longitudinal Study Participants and Members of the Public Towards Data Sharing for Secondary Research: A Survey Based Approach Supplemental material, sj-docx-1-jre-10.1177_15562646261452724 for Attitudes of Longitudinal Study Participants and Members of the Public Towards Data Sharing for Secondary Research: A Survey Based Approach by Nicola Howe, Thomas Chadwick, Dorothy Newbury-Birch and Elaine McColl in Journal of Empirical Research on Human Research Ethics

sj-docx-2-jre-10.1177_15562646261452724 - Supplemental material for Attitudes of Longitudinal Study Participants and Members of the Public Towards Data Sharing for Secondary Research: A Survey Based Approach Supplemental material, sj-docx-2-jre-10.1177_15562646261452724 for Attitudes of Longitudinal Study Participants and Members of the Public Towards Data Sharing for Secondary Research: A Survey Based Approach by Nicola Howe, Thomas Chadwick, Dorothy Newbury-Birch and Elaine McColl in Journal of Empirical Research on Human Research Ethics
