# Supplementary material for: Attitudes of Longitudinal Study Participants and Members of the Public Towards Data Sharing for Secondary Research: A Survey Based Approach
Source: J Empir Res Hum Res Ethics. 2026 Jun 3;21(4):253–74. doi: 10.1177/15562646261452724 (PMC13554376; doi:10.1177/15562646261452724)
Supplement: Supplementary file 1 [file sj-docx-1-jre-10.1177_15562646261452724.docx]

supplementary online documents

Question 7a Which of the above statements is of MOST concern to you?

| Response: | Number of respondents (%) | | | | | | | |
| --- | --- | --- | --- | --- | --- | --- | --- | --- |
|  | **All participants** | | **ACONF** | | **ALSPAC** | | **PPI Groups** | |
| a) If I could still be identified in the data | 715 | 46.1 | 110 | 32.5 | 580 | 50.4 | 25 | 40.3 |
| b) If my data could be used in research I don’t approve of | 154 | 9.9 | 41 | 12.1 | 102 | 8.9 | 11 | 17.7 |
| c) If my data could be stolen | 325 | 21.0 | 93 | 27.4 | 226 | 19.7 | 6 | 9.7 |
| d) If my data could be used for making a profit e.g., advertising instead of research | 152 | 9.8 | 43 | 12.7 | 102 | 8.9 | 7 | 11.3 |
| e) If it would be embarrassing if my data was linked back to me | 84 | 5.4 | 14 | 4.1 | 67 | 5.8 | 3 | 4.8 |
| f) If people could misinterpret the data and come to the wrong conclusions | 48 | 3.1 | 19 | 5.6 | 25 | 2.2 | 4 | 6.5 |
| g) If the original research team didn’t get credit for collecting the data | 23 | 1.5 | 2 | 0.6 | 21 | 1.8 | 0 | 0 |
| h) If it stopped researchers doing their own original research | 50 | 3.2 | 17 | 5.0 | 27 | 2.4 | 6 | 9.7 |
| TOTAL | 1,551 | 92.1 | 339 | 85.8 | 1,150 | 93.8 | 62 | 98.4 |

Question 8 How likely would you be to give permission for your data to be shared for the following reasons?

|  | Response: | Number of respondents (%) | | | | | | | |
| --- | --- | --- | --- | --- | --- | --- | --- | --- | --- |
|  |  | **All respondents** | | **ACONF** | | **ALSPAC** | | **PPI groups** | |
| To do research in a University | Very likely | 974 | 62.6 | 231 | 67.2 | 702 | 61.0 | 41 | 67.2 |
|  | Somewhat likely | 467 | 30.0 | 86 | 25.0 | 364 | 31.7 | 17 | 27.9 |
|  | Neither likely or unlikely | 69 | 4.4 | 21 | 6.1 | 47 | 4.1 | 1 | 1.6 |
|  | Somewhat unlikely | 27 | 1.7 | 3 | 0.9 | 23 | 2.0 | 1 | 1.6 |
|  | Very unlikely | 18 | 1.2 | 3 | 0.9 | 14 | 1.2 | 1 | 1.6 |
|  | Total | 1,555 | 100 | 344 | 100 | 1,150 | 100 | 61 | 100 |
| To do research in a hospital | Very likely | 1,119 | 72.2 | 266 | 77.6 | 811 | 70.8 | 42 | 68.9 |
|  | Somewhat likely | 371 | 24.0 | 61 | 17.8 | 294 | 25.7 | 16 | 26.2 |
|  | Neither likely or unlikely | 24 | 1.6 | 10 | 2.9 | 13 | 1.1 | 1 | 1.6 |
|  | Somewhat unlikely | 19 | 1.2 | 3 | 0.9 | 16 | 1.4 | 0 | 0 |
|  | Very unlikely | 16 | 1.0 | 3 | 0.9 | 11 | 1.0 | 2 | 3.3 |
|  | Total | 1,549 | 100 | 343 | 100 | 1,145 | 100 | 61 | 100 |
| To help a pharmaceutical company do research | Very likely | 559 | 36.0 | 122 | 35.6 | 423 | 36.9 | 14 | 23.0 |
|  | Somewhat likely | 615 | 39.6 | 129 | 37.6 | 459 | 40.0 | 27 | 44.3 |
|  | Neither likely or unlikely | 205 | 13.2 | 53 | 15.5 | 142 | 12.4 | 10 | 16.4 |
|  | Somewhat unlikely | 122 | 7.9 | 31 | 9.0 | 86 | 7.5 | 5 | 8.2 |
|  | Very unlikely | 51 | 3.3 | 8 | 2.3 | 38 | 3.3 | 5 | 8.2 |
|  | Total | 1,552 | 100 | 343 | 100 | 1,148 | 100 | 61 | 100 |
| To help the government study health problems | Very likely | 552 | 35.6 | 121 | 35.5 | 406 | 35.4 | 25 | 41.0 |
|  | Somewhat likely | 606 | 39.1 | 134 | 39.3 | 450 | 39.2 | 22 | 36.1 |
|  | Neither likely or unlikely | 201 | 13.0 | 50 | 14.7 | 145 | 12.6 | 6 | 9.8 |
|  | Somewhat unlikely | 126 | 8.1 | 22 | 6.5 | 99 | 8.6 | 5 | 8.2 |
|  | Very unlikely | 64 | 4.1 | 14 | 4.1 | 47 | 4.1 | 3 | 4.9 |
|  | Total | 1,549 | 100 | 341 | 100 | 1,147 | 100 | 61 | 100 |
| To inform the public about a health issue. | Very likely | 742 | 47.8 | 171 | 50.2 | 537 | 46.7 | 34 | 55.7 |
|  | Somewhat likely | 554 | 35.7 | 113 | 33.1 | 424 | 36.9 | 17 | 27.9 |
|  | Neither likely or unlikely | 170 | 11.0 | 41 | 12.0 | 123 | 10.7 | 6 | 9.8 |
|  | Somewhat unlikely | 59 | 3.8 | 13 | 3.8 | 44 | 3.8 | 2 | 3.3 |
|  | Very unlikely | 26 | 1.7 | 3 | 0.9 | 21 | 1.8 | 2 | 3.3 |
|  | Total | 1,551 | 100 | 341 | 100 | 1,149 | 100 | 61 | 100 |
| To help students get data for projects | Very likely | 491 | 31.7 | 110 | 32.5 | 355 | 30.9 | 26 | 42.6 |
|  | Somewhat likely | 578 | 37.3 | 145 | 42.5 | 414 | 36.1 | 19 | 31.2 |
|  | Neither likely or unlikely | 294 | 19.0 | 56 | 16.5 | 229 | 20.0 | 9 | 14.8 |
|  | Somewhat unlikely | 134 | 8.7 | 19 | 5.6 | 109 | 9.5 | 6 | 9.8 |
|  | Very unlikely | 51 | 3.3 | 9 | 2.7 | 41 | 3.6 | 1 | 1.6 |
|  | Total | 1,548 | 100 | 339 | 100 | 1,148 | 100 | 61 | 100 |

Question 9: Below is a list of potential benefits of sharing. Which of these make you feel more positive about data sharing?

| Response: | Number of respondents (%) | | | | | | | |
| --- | --- | --- | --- | --- | --- | --- | --- | --- |
|  | **All respondents** | | **ACONF** | | **ALSPAC** | | **PPI groups** | |
| Researchers can check each other’s results and conclusions, making science more open. | 1,149 | 68.2 | 274 | 69.4 | 863 | 70.4 | 51 | 81.0 |
| Rarer diseases and conditions can be studied more easily using combined data, without having to wait for more studies. | 1,374 | 81.6 | 313 | 79.2 | 1,051 | 85.7 | 53 | 84.1 |
| Researchers can get quicker answers to scientific questions using data already collected. | 1,197 | 71.1 | 286 | 72.5 | 900 | 73.4 | 52 | 82.5 |
| Researchers can get the most out of participant’s contribution (data) to their studies. | 1,006 | 59.7 | 243 | 61.5 | 752 | 61.3 | 52 | 82.5 |
| I can contribute to more research that affects me or my family. | 1,029 | 61.1 | 236 | 59.8 | 787 | 64.2 | 57 | 90.5 |

Question 10: Would any of the following motivate you to allow your data to be shared?

|  | Response: | Number of respondents (%) | | | | | | | |
| --- | --- | --- | --- | --- | --- | --- | --- | --- | --- |
|  |  | **All respondents** | | **ACONF** | | **ALSPAC** | | **PPI groups** | |
| Assured anonymity of the data shared | Yes | 1,442 | 93.3 | 308 | 92.2 | 1,078 | 93.7 | 56 | 91.8 |
|  | No | 30 | 1.9 | 13 | 3.9 | 16 | 1.4 | 1 | 1.6 |
|  | Not sure | 74 | 4.8 | 13 | 3.9 | 57 | 5.0 | 4 | 6.6 |
|  | Total | 1,546 | 100 | 334 | 100 | 1,151 | 100 | 61 | 100 |
| Understanding exactly how the data will be used | Yes | 1,340 | 86.8 | 278 | 83.2 | 1,012 | 88.0 | 50 | 83.3 |
|  | No | 102 | 6.6 | 26 | 7.8 | 71 | 6.2 | 5 | 8.3 |
|  | Not sure | 102 | 6.6 | 30 | 9.0 | 67 | 5.8 | 5 | 8.3 |
|  | Total | 1,544 | 100 | 334 | 100 | 1,150 | 100 | 60 | 100 |
| Knowing exactly who will access the data | Yes | 1,300 | 84.3 | 285 | 85.3 | 966 | 84.2 | 49 | 81.7 |
|  | No | 112 | 7.3 | 22 | 6.6 | 82 | 7.1 | 8 | 13.3 |
|  | Not sure | 130 | 8.4 | 27 | 8.1 | 100 | 8.7 | 3 | 5.0 |
|  | Total | 1,542 | 100 | 334 | 100 | 1,148 | 100 | 60 | 100 |
| Chance to understand my own condition better | Yes | 1,050 | 68.3 | 264 | 80.5 | 739 | 64.3 | 47 | 78.3 |
|  | No | 60 | 3.9 | 30 | 9.2 | 24 | 2.1 | 6 | 10.0 |
|  | Not sure | 93 | 6.1 | 34 | 10.4 | 52 | 4.5 | 7 | 11.7 |
|  | Not Applicable | 334 | 21.7 | - | - | 334 | 29.1 | - | - |
|  | Total | 1,537 | 100 | 328 | 100 | 1,149 | 100 | 60 | 100 |
| Chance to help others by contributing to research | Yes | 1,441 | 92.9 | 326 | 95.88 | 1.058 | 92.0 | 57 | 93.44 |
|  | No | 27 | 1.7 | 2 | 0.59 | 24 | 2.09 | 1 | 1.64 |
|  | Not sure | 83 | 5.4 | 12 | 3.53 | 68 | 5.91 | 3 | 4.92 |
|  | Total | 1,551 | 100 | 340 | 100 | 1,150 | 100 | 61 | 100 |

Question 11: Imagine that the researcher from the study you took part in wants to share your data with other researchers. How willing would you be for them to share anonymised details of your:

|  | Response: | Number of respondents (%) | | | | | | | |
| --- | --- | --- | --- | --- | --- | --- | --- | --- | --- |
|  |  | All respondents | | ACONF | | ALSPAC | | PPI groups | |
| Age | Not at all willing | 11 | 0.7 | 2 | 0.6 | 8 | 0.7 | 1 | 1.6 |
|  | Not very willing | 17 | 1.1 | 5 | 1.5 | 10 | 0.9 | 2 | 3.2 |
|  | Not sure | 42 | 2.7 | 12 | 3.5 | 28 | 2.4 | 2 | 3.2 |
|  | Willing | 595 | 38.3 | 133 | 39.1 | 444 | 38.6 | 18 | 29.0 |
|  | Very willing | 888 | 57.2 | 188 | 55.3 | 661 | 57.4 | 39 | 62.6 |
|  | Total | 1,553 | 100 | 340 | 100 | 1,151 | 100 | 62 | 100 |
| Gender | Not at all willing | 12 | 0.8 | 1 | 0.3 | 10 | 0.9 | 1 | 1.6 |
|  | Not very willing | 12 | 0.8 | 3 | 0.9 | 9 | 0.8 | 0 | 0 |
|  | Not sure | 37 | 2.4 | 8 | 2.4 | 27 | 2.4 | 2 | 3.2 |
|  | Willing | 590 | 38.1 | 137 | 40.5 | 434 | 37.8 | 19 | 30.7 |
|  | Very willing | 898 | 58.0 | 189 | 55.9 | 669 | 58.2 | 40 | 64.5 |
|  | Total | 1,549 | 100 | 338 | 100 | 1,149 | 100 | 62 | 100 |
| Education | Not at all willing | 27 | 1.8 | 3 | 0.9 | 22 | 1.9 | 2 | 3.3 |
|  | Not very willing | 47 | 3.0 | 4 | 1.2 | 42 | 3.7 | 1 | 1.6 |
|  | Not sure | 115 | 7.4 | 21 | 6.2 | 89 | 7.8 | 5 | 8.2 |
|  | Willing | 583 | 37.7 | 135 | 39.9 | 431 | 37.5 | 17 | 27.9 |
|  | Very willing | 775 | 50.1 | 175 | 51.8 | 564 | 49.1 | 36 | 59.0 |
|  | Total | 1,547 | 100 | 338 | 100 | 1,148 | 100 | 61 | 100 |
| Employment | Not at all willing | 59 | 3.8 | 4 | 1.2 | 53 | 4.6 | 2 | 3.2 |
|  | Not very willing | 82 | 5.3 | 7 | 2.1 | 72 | 6.3 | 3 | 4.8 |
|  | Not sure | 186 | 12.0 | 22 | 6.5 | 158 | 13.8 | 6 | 9.7 |
|  | Willing | 519 | 33.5 | 137 | 40.4 | 363 | 31.6 | 19 | 30.7 |
|  | Very willing | 703 | 45.4 | 169 | 49.9 | 502 | 43.7 | 32 | 51.6 |
|  | Total | 1,549 | 100 | 339 | 100 | 1,148 | 100 | 62 | 100 |
| Height & weight | Not at all willing | 20 | 1.3 | 4 | 1.2 | 15 | 1.3 | 1 | 1.6 |
|  | Not very willing | 34 | 2.2 | 7 | 2.1 | 25 | 2.2 | 2 | 3.2 |
|  | Not sure | 97 | 6.3 | 10 | 2.9 | 78 | 6.8 | 9 | 14.5 |
|  | Willing | 601 | 38.7 | 143 | 42.1 | 443 | 38.5 | 15 | 24.2 |
|  | Very willing | 800 | 51.6 | 176 | 51.8 | 589 | 51.2 | 35 | 56.5 |
|  | Total | 1,552 | 100 | 340 | 100 | 1,150 | 100 | 62 | 100 |
| Mental health | Not at all willing | 35 | 2.3 | 3 | 0.9 | 31 | 2.7 | 1 | 1.6 |
|  | Not very willing | 74 | 4.8 | 9 | 2.7 | 61 | 5.3 | 4 | 6.5 |
|  | Not sure | 188 | 12.2 | 30 | 8.9 | 150 | 13.1 | 8 | 12.9 |
|  | Willing | 545 | 35.2 | 136 | 40.2 | 393 | 34.3 | 16 | 25.8 |
|  | Very willing | 705 | 45.6 | 160 | 47.3 | 512 | 44.6 | 33 | 53.2 |
|  | Total | 1,547 | 100 | 338 | 100 | 1,147 | 100 | 62 | 100 |
| Cancers | Not at all willing | 24 | 1.6 | 1 | 0.3 | 22 | 1.9 | 1 | 1.6 |
|  | Not very willing | 31 | 2.0 | 5 | 1.5 | 24 | 2.1 | 2 | 3.3 |
|  | Not sure | 145 | 9.4 | 27 | 8.0 | 113 | 9.8 | 5 | 8.2 |
|  | Willing | 555 | 35.9 | 129 | 38.2 | 409 | 35.6 | 17 | 27.9 |
|  | Very willing | 792 | 51.2 | 176 | 52.1 | 580 | 50.5 | 36 | 59.0 |
|  | Total | 1,547 | 100 | 338 | 100 | 1,148 | 100 | 61 | 100 |
| HIV infection | Not at all willing | 50 | 3.3 | 10 | 3.0 | 38 | 3.3 | 2 | 3.2 |
|  | Not very willing | 47 | 3.1 | 6 | 1.8 | 38 | 3.3 | 3 | 4.8 |
|  | Not sure | 209 | 13.6 | 52 | 15.5 | 150 | 13.2 | 7 | 11.3 |
|  | Willing | 505 | 32.9 | 118 | 35.2 | 371 | 32.5 | 16 | 25.8 |
|  | Very willing | 726 | 47.2 | 149 | 44.5 | 543 | 47.6 | 34 | 54.8 |
|  | Total | 1,537 | 100 | 335 | 100 | 1,140 | 100 | 62 | 100 |
| Other diseases or conditions | Not at all willing | 22 | 1.4 | 1 | 0.3 | 20 | 1.7 | 1 | 1.6 |
|  | Not very willing | 29 | 1.9 | 6 | 1.8 | 21 | 1.8 | 2 | 3.2 |
|  | Not sure | 177 | 11.4 | 38 | 11.3 | 132 | 11.5 | 7 | 11.3 |
|  | Willing | 568 | 36.7 | 130 | 38.6 | 423 | 36.8 | 15 | 24.2 |
|  | Very willing | 753 | 48.6 | 162 | 48.1 | 554 | 48.2 | 37 | 59.7 |
|  | Total | 1,549 | 100 | 337 | 100 | 1,150 | 100 | 62 | 100 |
| Family history of disease | Not at all willing | 26 | 1.7 | 3 | 0.9 | 22 | 1.9 | 1 | 1.6 |
|  | Not very willing | 40 | 2.6 | 4 | 1.2 | 35 | 3.0 | 1 | 1.6 |
|  | Not sure | 150 | 9.7 | 18 | 5.3 | 124 | 10.8 | 8 | 12.9 |
|  | Willing | 592 | 38.2 | 142 | 42.0 | 430 | 37.4 | 20 | 32.3 |
|  | Very willing | 743 | 47.9 | 171 | 50.6 | 540 | 46.9 | 32 | 51.6 |
|  | Total | 1,551 | 100 | 338 | 100 | 1,151 | 100 | 62 | 100 |
| Reproductive health | Not at all willing | 31 | 2.0 | 3 | 0.9 | 27 | 2.4 | 1 | 1.7 |
|  | Not very willing | 49 | 3.2 | 6 | 1.8 | 41 | 3.6 | 2 | 3.3 |
|  | Not sure | 143 | 9.3 | 27 | 8.0 | 107 | 9.3 | 9 | 15.0 |
|  | Willing | 587 | 38.0 | 140 | 41.4 | 431 | 37.5 | 16 | 26.7 |
|  | Very willing | 736 | 47.6 | 162 | 47.9 | 542 | 47.2 | 32 | 53.3 |
|  | Total | 1,546 | 100 | 338 | 100 | 1,148 | 100 | 60 | 100 |
| Medications being taken | Not at all willing | 27 | 1.7 | 1 | 0.3 | 25 | 2.2 | 1 | 1.6 |
|  | Not very willing | 45 | 2.9 | 5 | 1.5 | 39 | 3.4 | 1 | 1.6 |
|  | Not sure | 119 | 7.7 | 18 | 5.3 | 97 | 8.4 | 4 | 6.5 |
|  | Willing | 590 | 38.0 | 135 | 39.8 | 436 | 37.9 | 19 | 30.7 |
|  | Very willing | 770 | 49.7 | 180 | 53.1 | 553 | 48.1 | 37 | 59.7 |
|  | Total | 1,551 | 100 | 339 | 100 | 1,150 | 100 | 62 | 100 |
| Smoking behaviour | Not at all willing | 24 | 1.6 | 2 | 0.6 | 21 | 1.8 | 1 | 1.6 |
|  | Not very willing | 28 | 1.8 | 3 | 0.9 | 24 | 2.1 | 1 | 1.6 |
|  | Not sure | 67 | 4.3 | 12 | 3.6 | 53 | 4.6 | 2 | 3.2 |
|  | Willing | 580 | 37.5 | 131 | 38.9 | 430 | 37.5 | 19 | 30.7 |
|  | Very willing | 846 | 54.8 | 189 | 56.1 | 618 | 53.9 | 39 | 62.9 |
|  | Total | 1,545 | 100 | 337 | 100 | 1,146 | 100 | 62 | 100 |
| Alcohol use | Not at all willing | 23 | 1.5 | 1 | 0.3 | 21 | 1.8 | 1 | 1.6 |
|  | Not very willing | 30 | 1.9 | 4 | 1.2 | 26 | 2.3 | 0 | 0 |
|  | Not sure | 67 | 4.3 | 16 | 4.7 | 49 | 4.3 | 2 | 3.2 |
|  | Willing | 599 | 38.8 | 131 | 38.8 | 446 | 38.9 | 22 | 35.5 |
|  | Very willing | 827 | 53.5 | 186 | 55.0 | 604 | 52.7 | 37 | 59.7 |
|  | Total | 1,546 | 100 | 338 | 100 | 1,146 | 100 | 62 | 100 |
| Illegal drug use | Not at all willing | 50 | 3.2 | 5 | 1.5 | 43 | 3.8 | 2 | 3.3 |
|  | Not very willing | 47 | 3.1 | 6 | 1.8 | 40 | 3.5 | 1 | 1.6 |
|  | Not sure | 141 | 9.1 | 31 | 9.3 | 109 | 9.5 | 1 | 1.6 |
|  | Willing | 522 | 33.9 | 117 | 34.9 | 386 | 33.7 | 19 | 31.2 |
|  | Very willing | 782 | 50.7 | 176 | 52.5 | 568 | 49.6 | 38 | 62.3 |
|  | Total | 1,542 | 100 | 335 | 100 | 1,146 | 100 | 61 | 100 |

Question 13: What information would you like to see on the consent form before you agree to share your data?

|  | Response: | Number of respondents (%) | | | | | | | |
| --- | --- | --- | --- | --- | --- | --- | --- | --- | --- |
|  |  | **All respondents** | | **ACONF** | | **ALSPAC** | | **PPI groups** | |
| Explain that my data may be shared | Yes | 1,471 | 97.4 | 321 | 96.4 | 1,089 | 97.5 | 61 | 100 |
|  | No | 24 | 1.6 | 4 | 1.2 | 20 | 1.8 | 0 | 0 |
|  | Not sure | 16 | 1.6 | 8 | 2.4 | 8 | 0.7 | 0 | 0 |
|  | Total | 1,511 | 100 | 333 | 100 | 1,117 | 100 | 61 | 100 |
| HOW the researchers will protect (anonymise) my identity. | Yes | 1,416 | 94.0 | 295 | 89.4 | 1,064 | 95.3 | 57 | 93.4 |
|  | No | 65 | 4.3 | 25 | 7.6 | 36 | 3.2 | 4 | 6.6 |
|  | Not sure | 26 | 1.7 | 10 | 3.0 | 16 | 1.4 | 0 | 0 |
|  | Total | 1,507 | 100 | 330 | 100 | 1,116 | 100 | 61 | 100 |
| Explanation of WHO might benefit from using my data | Yes | 1,361 | 90.5 | 291 | 88.7 | 1,017 | 91.1 | 53 | 83.3 |
|  | No | 91 | 6.1 | 25 | 7.6 | 62 | 5.6 | 4 | 6.7 |
|  | Not sure | 52 | 3.5 | 12 | 3.7 | 37 | 3.3 | 3 | 5.0 |
|  | Total | 1,504 | 100 | 328 | 100 | 1,116 | 100 | 60 | 100 |
| Details of WHERE the data will be stored. | Yes | 1,138 | 75.8 | 238 | 73.2 | 852 | 76.3 | 48 | 80.0 |
|  | No | 254 | 16.9 | 56 | 17.2 | 188 | 16.9 | 10 | 16.7 |
|  | Not sure | 109 | 7.3 | 31 | 9.5 | 76 | 6.8 | 2 | 3.3 |
|  |  | 1,501 | 100 | 325 | 100 | 1,116 | 100 | 60 | 100 |
| Details of HOW the data will be stored. | Yes | 1,122 | 75.0 | 240 | 74.3 | 837 | 75.1 | 45 | 76.3 |
|  | No | 261 | 17.5 | 57 | 17.7 | 193 | 17.3 | 11 | 18.6 |
|  | Not sure | 113 | 7.6 | 26 | 8.1 | 84 | 7.5 | 3 | 5.1 |
|  | Total | 1,496 | 100 | 323 | 100 | 1,114 | 100 | 59 | 100 |
| Details of WHO the data might be shared with. | Yes | 1,423 | 94.5 | 300 | 90.6 | 1,067 | 95.7 | 56 | 93.3 |
|  | No | 52 | 3.5 | 16 | 4.8 | 33 | 3.0 | 3 | 5.0 |
|  | Not sure | 31 | 2.1 | 15 | 4.5 | 15 | 1.4 | 1 | 1.7 |
|  | Total | 1,506 | 100 | 331 | 100 | 1,115 | 100 | 60 | 100 |

Question 16 Would you prefer to give consent separately for each type of organisation your data could be shared with?

| Response: | No. of respondents (%) | | | | | | | |
| --- | --- | --- | --- | --- | --- | --- | --- | --- |
|  | **All respondents** | | **ACONF** | | **ALSPAC** | | **PPI groups** | |
| Yes | 907 | 60.1 | 182 | 54.8 | 688 | 61.5 | 37 | 61.7 |
| No | 403 | 26.7 | 115 | 34.6 | 271 | 24.2 | 17 | 28.3 |
| Not sure | 200 | 13.3 | 35 | 10.5 | 159 | 14.2 | 6 | 10.0 |
| Total | 1,510 | 100 | 332 | 100 | 1,118 | 100 | 60 | 100 |

Question 17: Do you think a register of participants willing to share their study data is a good idea?

| Response: | No. of respondents (%) | | | | | | | |
| --- | --- | --- | --- | --- | --- | --- | --- | --- |
|  | **All respondents** | | **ACONF** | | **ALSPAC** | | **PPI groups** | |
| Yes | 961 | 63.8 | 218 | 66.7 | 706 | 63.1 | 37 | 60.7 |
| No | 195 | 12.9 | 33 | 10.1 | 151 | 13.5 | 11 | 18.0 |
| Not sure | 351 | 23.3 | 76 | 23.2 | 262 | 23.4 | 13 | 21.3 |
| Total | 1,507 | 100 | 327 | 100 | 1,119 | 100 | 61 | 100 |

Question 18: Would you be willing to be named on it?

| Response: | No. of respondents (%) | | | | | | | |
| --- | --- | --- | --- | --- | --- | --- | --- | --- |
|  | **All respondents** | | **ACONF** | | **ALSPAC** | | **PPI groups** | |
| Yes | 763 | 50.7 | 165 | 50.5 | 565 | 50.5 | 33 | 54.1 |
| No | 244 | 16.2 | 67 | 20.5 | 160 | 14.3 | 17 | 27.9 |
| Not sure | 499 | 33.1 | 95 | 29.1 | 393 | 35.2 | 11 | 18.0 |
| Total | 1,506 | 100 | 327 | 100 | 1,118 | 100 | 61 | 100 |

Question 20: If data has controlled access: Who do you think should give permission for data to be shared and used again?

| Response: | No. of respondents (%) | | | | | | | |
| --- | --- | --- | --- | --- | --- | --- | --- | --- |
|  | **All respondents** | | **ACONF** | | **ALSPAC** | | **PPI groups** | |
| The participants who took part should decide | 580 | 38.8 | 120 | 36.7 | 438 | 39.5 | 22 | 37.3 |
| The researcher(s) who collected it | 285 | 19.1 | 61 | 18.7 | 219 | 19.8 | 5 | 8.5 |
| The organisation where the original researcher(s) work | 349 | 23.3 | 95 | 29.1 | 236 | 21.3 | 18 | 30.5 |
| An independent committee | 138 | 9.2 | 30 | 9.2 | 99 | 8.9 | 9 | 15.3 |
| Other | 7 | 0.5 | 0 | 0 | 6 | 0.5 | 1 | 1.7 |
| Not sure | 136 | 9.1 | 21 | 6.4 | 111 | 10.0 | 4 | 6.8 |
| Total | 1,495 | 100 | 327 | 100 | 1,109 | 100 | 59 | 100 |

Question 21: Who do you think should ‘own’ the data collected during a study?

| Response: | No. of respondents (%) | | | | | | | |
| --- | --- | --- | --- | --- | --- | --- | --- | --- |
|  | **All respondents** | | **ACONF** | | **ALSPAC** | | **PPI groups** | |
| Me/the participants who took part | 831 | 49.4 | 208 | 52.7 | 616 | 50.2 | 7 | 11.1 |
| The researcher(s) who collected it | 761 | 45.2 | 133 | 33.7 | 624 | 50.9 | 4 | 6.4 |
| The organisation where the original researcher(s) work | 742 | 44.1 | 194 | 49.1 | 543 | 44.3 | 5 | 7.9 |
| Anyone who uses it | 26 | 1.5 | 6 | 1.5 | 20 | 1.6 | 0 | 0 |
| Whoever stores it | 30 | 1.8 | 6 | 1.5 | 24 | 2.0 | 0 | 0 |
| No one | 41 | 2.4 | 10 | 2.5 | 30 | 2.5 | 1 | 1.6 |
| Other | 5 | 0.3 | 1 | 0.3 | <5 | 0.3 | 0 | 0 |
| Not sure | 76 | 4.5 | 10 | 2.5 | 66 | 5.4 | 0 | 0 |

Do you think it is important that researchers using shared data give feedback telling participants how their data was used?

| Response: | No. of respondents (%) | | | | | | | |
| --- | --- | --- | --- | --- | --- | --- | --- | --- |
|  | **All respondents** | | **ACONF** | | **ALSPAC** | | **PPI groups** | |
| Yes | 1,226 | 81.9 | 244 | 74.9 | 929 | 83.7 | 53 | 86.9 |
| No | 126 | 8.4 | 48 | 14.7 | 74 | 6.7 | 4 | 6.6 |
| Not sure | 145 | 9.7 | 34 | 10.4 | 107 | 9.6 | 4 | 6.6 |
| Total | 1,497 | 100 | 326 | 100 | 1,110 | 100 | 61 | 100 |

Question 29: Do you have any further comments about data sharing or about this survey?

| **Nature of comments:** | **No. %** | |
| --- | --- | --- |
| Respondent would prefer conditional consent based upon secondary use or recipient | 42 | 26 |
| Respondent is happy with sharing as long as they can not be identified | 35 | 22 |
| The benefits of sharing/altruistic reasons for sharing | 18 | 11 |
| Other comments | 16 | 10 |
| Respondent would be interested in feedback on use of their data | 13 | 8 |
| Respondent has some reservations about sharing data | 13 | 8 |
| Respondent trusts researchers to make appropriate sharing decisions | 11 | 7 |
| General remarks about consent | 8 | 5 |
| Respondent has no concerns about data sharing | 5 | 3 |
| All comments | 161 | 100 |
