# Supplementary material for: Attitudes of Longitudinal Study Participants and Members of the Public Towards Data Sharing for Secondary Research: A Survey Based Approach
Source: J Empir Res Hum Res Ethics. 2026 Jun 3;21(4):253–74. doi: 10.1177/15562646261452724 (PMC13554376; doi:10.1177/15562646261452724)
Supplement: Supplementary file 2 [file sj-docx-2-jre-10.1177_15562646261452724.docx]

Your views on sharing data from clinical trials and health research studies

Start of Block: Patient Information Sheet

 **Your views on sharing data from clinical trials and health research studies**
 
**About this survey:**
 
 We are interested in how members of the public, or people who have taken part in research, feel about what researchers do with their data at the end of the study.    When participants take part in a research study, they are asked to sign a consent form. The consent form checks that the participant is happy to have their data included in the research study. Sometimes the consent form asks permission to share the data with other researchers at the end of the study.  We would like to understand how participants feel about researchers sharing their data with other researchers.   We hope that by understanding participant’s preferences for data sharing, researchers could change the way participants are told about data sharing, or how they are asked to consent to share data. 
 
**What does taking part involve?**
 
 Taking part in the study involves completing the attached survey.    It should take approximately 20 minutes to complete this survey. You can also save your answers and continue the survey at another time.   
  
   Your survey responses will be given a study number and saved in a database. The survey data will be analysed with the responses from the other people completing the survey.    There are no wrong or right answers - we are just interested in your opinion and any relevant experiences. 
 
**Risks and Benefits:**
 
 Participating in this study has no direct benefits to you, although you may find it rewarding to contribute to research.   We will take steps to ensure that your survey responses remain confidential. Your data will only be identified by a study number and your data will be stored securely.   This study was approved by the Faculty of Medical Sciences Research Ethics Committee, part of Newcastle University Research Ethics Committee. This committee contains members who are internal to the Faculty, as well as one external member. This study was reviewed by members of the committee, who must provide impartial advice and avoid significant conflicts of interests.  

   
**Consent:**
 
 By completing this survey, you are giving your informed consent to take part.   Completing this survey will not affect the treatment you will receive in any study or trial in which you are already involved. It won’t affect what happens to data from any studies or trials you may have been involved in.   The results of this survey may be presented at scientific meetings or published in scientific journals. The de-identified survey data may be shared with other researchers if it is requested, and if approval is granted.    The survey data may be shared with the original study team who contacted you to take part (if applicable). 
 
    **For more information, or if you have any concerns about the study please contact:**
 

  
  
   End of Block: Patient Information Sheet

Start of Block: Questions about taking part in research

Survey Background
 This survey asks your opinions on sharing of data collected in a clinical trial or health research study.
 A clinical trial is a medical study in which participants receive treatment according to a research plan. These treatments could be medicines; medical procedures; or attempts to change participant’s’ behaviour, such as their diet. Clinical trials may be designed to study a new treatment or to learn more about the safety and effectiveness of treatments that are already in use.
  A Health research study is not a medical trial but may investigate the effects of an intervention (e.g., giving health information) on participant’s health. Some studies may collect data from participants over a long time period in order to study their health. Examples could include studying participants' diet, weight, or levels of exercise. 
 Data sharing means allowing other researchers (who aren’t part of the original research team) to see and use study data for further research. Personal identifiers (like names and birthdates) should be removed so that the data is anonymous. Data could be shared with other researchers or companies developing medical products.   This survey will use the word ‘study’ to mean both clinical trial or **health re**search study.

Q1
**Questions about taking part in research**
 
**Have any of the following ever taken part in a health research study?**
 
***(select all that apply)***

- You (1)
- Your child (2)
- ⊗Neither (3)
- ⊗Not sure (4)

Display This Question:

If Q1 = You

Q2a
**Was YOUR participation in the study as:**
 
***(please select one)***

- A person who had the health condition being studied? (1)
- A healthy volunteer? (2)
- A person who is at risk of developing the health condition being studied? (3)
- Not sure (4)

Display This Question:

If Q1 = Your child

Q2b **Was YOUR CHILD'S participation in the study as:

 *(please select one)***

- A child who had the health condition being studied? (1)
- A healthy volunteer? (2)
- A child who is at risk of developing the health condition being studied? (3)
- Not sure (4)

Display This Question:

If Q1 = You

Q3a
**What was the experience of taking part in a study like for YOU?**
 
***(please select one)***

- Very positive (1)
- Positive (2)
- Neither positive or negative (3)
- Negative (4)
- Very negative (5)
- Not applicable (6)
- Not sure (7)

Display This Question:

If Q1 = Your child

Q3b **What was the experience of taking part in a study like for YOUR CHILD?**

 ***(please select one)***

- Very positive (1)
- Positive (2)
- Neither positive nor negative (3)
- Negative (4)
- Very negative (5)
- Not applicable (6)
- Not sure (7)

End of Block: Questions about taking part in research

Start of Block: Questions about Data Sharing

Display This Question:

If Q1 = Not sure

Or Q1 = Neither

Instructions for completion
  If you have NOT taken part in a health research study, please answer the questions as if you had taken part or are going to take part- think about your study data being shared.

| Page Break |  |
| --- | --- |

End of Block: Questions about Data Sharing

Start of Block: Questoins 5 and 6

**Questions about Data Sharing**
Remember, sharing study data means sharing information about each individual research participant- for example, age, health conditions, and response to the treatment being tested-not just the overall results of the study. 
The data will be anonymised- personal identifiers (like names and birth dates) would be removed. Data could be shared with other researchers or with companies developing medical products.

Q5 **How concerned would you be if you knew data from a study that you were involved in was being shared?**

 ***(please select one)***

- Very concerned (1)
- Somewhat concerned (2)
- Not very concerned (3)
- Not at all concerned (4)
- Not sure (5)
- Depends who it is shared with (6)

Q6 How concerned would you be if you knew data was being shared with:

|  | Very concerned (1) | Somewhat concerned (2) | Not very concerned (3) | Not at all concerned (4) | Not sure (5) |
| --- | --- | --- | --- | --- | --- |
| Researchers at the same organisation where your data was collected (1) |  |  |  |  |  |
| Researchers at a pharmaceutical company, e.g. for developing new medicines (2) |  |  |  |  |  |
| Researchers at a university (3) |  |  |  |  |  |
| Researchers at a hospital (4) |  |  |  |  |  |
| Researchers in another country (5) |  |  |  |  |  |
| A charity or not for profit organisation (6) |  |  |  |  |  |
| The government (7) |  |  |  |  |  |
| A student at a university (8) |  |  |  |  |  |
| On the internet for anyone to use (9) |  |  |  |  |  |

End of Block: Questoins 5 and 6

Start of Block: Q7

Q7 **If data from a study in which you were involved was being shared, how concerned would you be about the following?**

|  | Very concerned (1) | Somewhat concerned (2) | Not very concerned (3) | Not at all concerned (4) | Not sure (5) |
| --- | --- | --- | --- | --- | --- |
| a) If I could still be identified in the data (1) |  |  |  |  |  |
| b) If my data could be used in research I don’t approve of (2) |  |  |  |  |  |
| c) If my data could be stolen (3) |  |  |  |  |  |
| d) If my data could be used for making a profit e.g., advertising instead of research (4) |  |  |  |  |  |
| e) If it would be embarrassing if my data was linked back to me (5) |  |  |  |  |  |
| f) If people could misinterpret the data and come to the wrong conclusions (6) |  |  |  |  |  |
| g) If the original research team didn’t get credit for collecting the data (7) |  |  |  |  |  |
| h) If it stopped researchers doing their own original research (8) |  |  |  |  |  |

Q7a **Which of the above statements is of MOST concern to you?**
 
***(please select one, a-h)***

- a- If I could still be identified from the data (1)
- b- If my data could be used for research I don't approve of (2)
- c- If my data could be stolen (3)
- d- If my data could be used for making profit (4)
- e- If it would be embarrassing if my data was linked back to me (5)
- f- If people could misinterpret the data (6)
- g- If the original research team didn't get credit (7)
- h- If it stopped researchers doing original research (8)

End of Block: Q7

Start of Block: Q8

Q8 **How likely would you be to give permission for your data to be shared for the following reasons?**

|  | Very likely (1) | Somewhat likely (2) | Neither likely nor unlikely (3) | Somewhat unlikely (4) | Very unlikely (5) |
| --- | --- | --- | --- | --- | --- |
| To do research in a University (1) |  |  |  |  |  |
| To do research in a hospital (2) |  |  |  |  |  |
| To help a pharmaceutical company do research (3) |  |  |  |  |  |
| To help the government study health problems (4) |  |  |  |  |  |
| To inform the public about a health issue. (5) |  |  |  |  |  |
| To help students get data for projects (6) |  |  |  |  |  |

End of Block: Q8

Start of Block: Questions 9 and 10

Q9 **Below is a list of potential benefits of data sharing. Which of these make you feel more positive about data sharing?**   ***(please select all that apply)***

- Researchers can check each other’s results and conclusions, making science more open (1)
- Rarer diseases and conditions can be studied more easily using combined data, without having to wait for more studies. (2)
- Researchers can get quicker answers to scientific questions using data already collected. (3)
- Researchers can get the most out of participant’s contribution (data) to their studies. (4)
- I can contribute to more research that affects me or my family. (5)

Q10
**Would any of the following motivate you to allow your data to be shared?**

|  | Yes (1) | No (2) | Not sure (3) |
| --- | --- | --- | --- |
| Assured anonymity of the data shared (1) |  |  |  |
| Understanding exactly how the data will be used (2) |  |  |  |
| Knowing exactly who will access the data (3) |  |  |  |
| Chance to understand my own condition better (4) |  |  |  |
| Chance to help others by contributing to research (5) |  |  |  |

End of Block: Questions 9 and 10

Start of Block: how willing to share details of...

Q11
  **Imagine that the researcher from the study you took part in wants to share your data with other researchers.** 
 
**How willing would you be for them to share anonymised details of your:**

|  | Not at all willing (1) | Not very willing (2) | not sure (3) | Willing (4) | Very willing (5) |
| --- | --- | --- | --- | --- | --- |
| Age (Q11_1) |  |  |  |  |  |
| Gender (Q11_2) |  |  |  |  |  |
| Education (Q11_3) |  |  |  |  |  |
| Employment (Q11_4) |  |  |  |  |  |
| Height & weight (Q11_5) |  |  |  |  |  |
| Mental health (Q11_6) |  |  |  |  |  |
| Cancers (Q11_7) |  |  |  |  |  |
| HIV infection (Q11_8) |  |  |  |  |  |
| Other diseases or conditions (Q11_9) |  |  |  |  |  |
| Family history of disease (Q11_10) |  |  |  |  |  |
| Reproductive health (Q11_11) |  |  |  |  |  |
| Medications being taken (Q11_12) |  |  |  |  |  |
| Smoking behaviour (Q11_13) |  |  |  |  |  |
| Alcohol use (Q11_14) |  |  |  |  |  |
| Illegal drug use (Q11_15) |  |  |  |  |  |

End of Block: how willing to share details of...

Start of Block: Questions about consent

**Questions about Consent**   Researchers should let participants know on the consent form that their study data could be shared.   The consent form is signed by participants (or parents/guardians of participants) at the beginning of a study.

Q12 **How and when would you like to be asked to share your data?**
 
***(please select one)***

- Once, on the consent form for the original study (1)
- Every time it is shared, with the option for me to say no (2)
- Just let me know every time it is shared (3)
- There is no need to ask me, just share it (4)
- I have no preference (5)

Q13
**What information would you like to see on the consent form before you agree to share your data?** 
 
***(please tick yes, no or not sure for each statement)***

|  | Yes (1) | No (2) | Not sure (3) |
| --- | --- | --- | --- |
| Explain that my data may be shared (1) |  |  |  |
| HOW the researchers will protect (anonymise) my identity. (8) |  |  |  |
| Explanation of WHO might benefit from using my data (3) |  |  |  |
| Details of WHERE the data will be stored. (4) |  |  |  |
| Details of HOW the data will be stored. (5) |  |  |  |
| Details of WHO the data might be shared with. (6) |  |  |  |

Q13a **OR**

- None of the above would convince me to share my data (1)

End of Block: Questions about consent

Start of Block: questions about consent 2

Q14 **How important is it that you are informed on the consent form that your study data might be shared?

 *(please select one)***

- Very important (1)
- Somewhat important (2)
- Not very important (3)
- Not at all important (4)
- Not sure (5)

Q15 **If you knew your data might be shared, what effect would it have on you taking part in a study?

 *(please select one)***

- I would not take part at all (1)
- I’d be much less likely to take part (2)
- I’d be a bit more cautious about taking part (3)
- It would have no effect on my decision to take part (4)
- I’d be a bit more likely to take part (5)
- I’d be much more likely to take part (6)
- Not sure (7)

Q16 **Would you prefer to give consent separately for each type of organisation your data could be shared with?**   ***(For example, separate consent for within the NHS, within the university doing the original research, outside the university, private companies).***

- Yes (1)
- No (2)
- Not sure (3)

End of Block: questions about consent 2

Start of Block: Questions about register

Q17 **Do you think a register of participants willing to share their study data is a good idea?**

 ***(researchers could refer to the register instead of having to gain consent for each study the participant takes part in)***

- Yes (1)
- No (2)
- Not sure (3)

Q18 **If a register of participants who are willing to share their study data existed, would you be willing to be named on it?**

- Yes (1)
- No (2)
- Not sure (3)

End of Block: Questions about register

Start of Block: Questions about data storage

**Questions about data storage**   **Data can be stored with *controlled access* or *open access*.** 
 With **controlled access,** there is a formal request and approval process in place before data can be shared.   With **open access,** data can be accessed by anyone.

Q19 **How would you prefer your study data to be stored?

 *(please select one)***

- Open access (1)
- Controlled access (2)
- No preference (3)
- Not sure (4)

Q20 **If data has controlled access:**   **Who do you think should give permission for data to be shared and used again?**   ***(please select one)***

- The participants who took part should decide (1)
- The researcher(s) who collected it (2)
- The organisation where the original researcher(s) work (3)
- An independent committee (4)
- Other (5)
- Not sure (6)

End of Block: Questions about data storage

Start of Block: ownership and feedback

Q21 **Who do you think should ‘own’ the data collected during a study?**    ***(please tick all that apply)***

- Me/the participants who took part (1)
- The researcher(s) who collected it (2)
- The organisation where the original researcher(s) work (3)
- Anyone who uses it (4)
- Whoever stores it (5)
- No one (6)
- Other (7)
- Not sure (8)

Q22 **Do you think it is important that researchers using shared data give feedback telling participants how their data was used?**   ***(please tick one)***

- Yes (1)
- No (2)
- Not sure (3)

End of Block: ownership and feedback

Start of Block: Questions about you

Q23
 **Questions about you**
  
**What is your gender?**

- Male (1)
- Female (2)
- Other (3)
- Prefer not to say (4)

Q24 **Which age group do you belong to?**

- 18-24 (1)
- 25-44 (2)
- 45-64 (3)
- 65-74 (4)
- 75-84 (5)
- 85 and over (6)
- Prefer not to say (7)

Q25 **How would you describe your ethnicity?**

- White (1)
- Mixed/multiple ethnic group (2)
- Asian/Asian British (3)
- Black/African/Caribbean/Black British (4)
- Chinese (5)
- Other ethnic group (6)
- Prefer not to say (7)

Q26 **What is your highest level of educational achievement?**   ***(please tick one)***

- No qualifications (1)
- O Levels/CSE/GCSE or equivalent (2)
- AS/A Levels or equivalent (3)
- Degree (e.g., BA, BSc) or equivalent (4)
- Higher degree (e.g., MSc, PhD) or equivalent (5)
- Professional qualifications (e.g., nursing, accountancy, teaching) (6)
- Other (7)
- Prefer not to say (8)

Q27 **How would you describe your overall health at the moment?**   ***(please tick one)***

- Excellent (1)
- Good (2)
- Average (3)
- Poor (4)
- Very poor (5)
- Not sure (6)
- Prefer not to say (7)

Q28 **What is your postcode?**
 
***(this question is optional)***

________________________________________________________________

Q29 **Do you have any further comments about data sharing or about this survey?** (***if yes, please use the space below)***

________________________________________________________________

________________________________________________________________

________________________________________________________________

________________________________________________________________

________________________________________________________________

Q30 **Would you be interested in taking part in further research about data sharing, for example as part of a focus group or by taking part in an interview?**  

- If yes, you can contact the lead researcher by email:

End of Block: Questions about you
